# The contribution of penguin guano to the Southern Ocean iron pool

**DOI:** 10.1038/s41467-023-37132-5

**Published:** 2023-04-11

**Authors:** Oleg Belyaev, Erica Sparaventi, Gabriel Navarro, Araceli Rodríguez-Romero, Antonio Tovar-Sánchez

**Affiliations:** 1 Department of Ecology and Coastal Management, Institute of Marine Sciences of Andalusia (ICMAN), Spanish National Research Council (CSIC), 11510 Puerto Real, Cádiz Spain; 2 Department of Analytical Chemistry, Faculty of Marine and Environmental Sciences, Marine Research Institute (INMAR), University of Cádiz, 11510 Puerto Real, Cádiz Spain

**Keywords:** Element cycles, Element cycles, Marine chemistry

## Abstract

Iron plays a crucial role in the high-nutrient, low-chlorophyll Southern Ocean regions, promoting phytoplankton growth and enhancing atmospheric carbon sequestration. In this area, iron-rich Antarctic krill (*Euphausia superba*) and baleen whale species, which are among their main predators, play a large role in the recycling of iron. However, penguins have received limited attention despite their representing the largest seabird biomass in the southern polar region. Here, we use breeding site guano volumes estimated from drone images, deep learning-powered penguin census, and guano chemical composition to assess the iron export to the Antarctic waters from one of the most abundant penguin species, the Chinstrap penguin (*Pygoscelis antarcticus*). Our results show that these seabirds are a relevant contributor to the iron remobilization pool in the Southern Ocean. With an average guano concentration of 3 mg iron g^−1^, we estimate that the Chinstrap penguin population is recycling 521 tonnes iron yr^−1^, representing the current iron contribution half of the amount these penguins were able to recycle four decades ago, as they have declined by more than 50% since then.

## Abstract (web summary)

Here the authors show that Chinstrap penguins play a significant role in iron recycling, essential for phytoplankton growth and carbon sequestration, recycling yearly 521 tonnes of iron, half of what they did 40 years ago due to population decline.

## Introduction

In the Southern Ocean, several areas such as the Antarctic Circumpolar Current (ACC) or regional zones with seasonally driven iron (Fe) depletions (e.g., Ross Sea polynya) are considered high-nutrient, low-chlorophyll regions^1–3^, where phytoplankton growth is limited by Fe availability^4–7^. Despite this limitation, this oceanic region is one of the major sinks of anthropogenic carbon dioxide (CO_2_)^8–10^, removing an estimated 10 × 10^13 ^kg carbon (C) from the pelagic zone each year^11–14^. Contrary to the relatively well understood physical processes that mediate C acquisition, the biological pump remains poorly described due to the high spatial and temporal variability of mechanisms such as uptake, remineralization, or export of key elements, like Fe^15^. The sources of these bioactive elements that sustain the biological pump have shown to depend on numerous non-localized factors, such as atmospheric dust deposition^16,17^, nearshore sediment input^18^, ice melting^19–21^, hydrothermal vents^22,23^ or upwelling^24^, among others. However, it is estimated that the magnitude of the contribution of biogenic sources to the enrichment of the southern pelagic regions is similar to that of non-biogenic inputs^25–27^.

The recycling of the biogenic Fe in the upper ocean layer has previously shown to be driven by Antarctic krill (*Euphausia superba*), a key Fe rich species in the Southern Ocean ecosystem^25,26^, and several whale species mainly via excretion products^25,28–30^. However, information about the role of seabirds, especially penguins as a source of Fe remains limited. Unlike whales, that roam free in northern latitude waters throughout their life stages, Antarctic penguins, especially the Pygoscelis genus (i.e., Chinstrap, Adélie and Papua), are restricted to the Southern Ocean^31^, breeding throughout the austral summer and foraging during the rest of the year. They reach the ice borders near the ACC in winter, where their contribution to Fe-depleted regions could be more significant. In the same way as occurs with non-biogenic Fe fertilization mechanisms^32^, it is possible to infer that biogenic Fe released during the migratory routes from the Antarctic Peninsula to vicinity of the ACC could have a similar distribution behaviour. In addition, the Chinstrap penguins feed almost exclusively (>90% of their diet) on krill^33–36^; and this feeding takes place within the upper 100 m of the water column, where primary production and photosynthetic fixation of carbon by phytoplankton occurs^37^. Furthermore, their significant abundance leads to the accumulation of high densities of Fe-containing guano in the coastal ecosystem, reaching concentrations of up to 3 × 10^3^ times higher than the background in the waters surrounding the rookeries^38^. These facts ultimately suggest that penguins could be playing a fundamental role in the Southern Ocean through Fe recycling^39–42^. Moreover, quantifying their role in the Antarctic ecosystem is even more relevant when overall declining trends in specific Antarctic penguin species populations have been shown during the last four decades^43–46^.

**Fig. 1:**
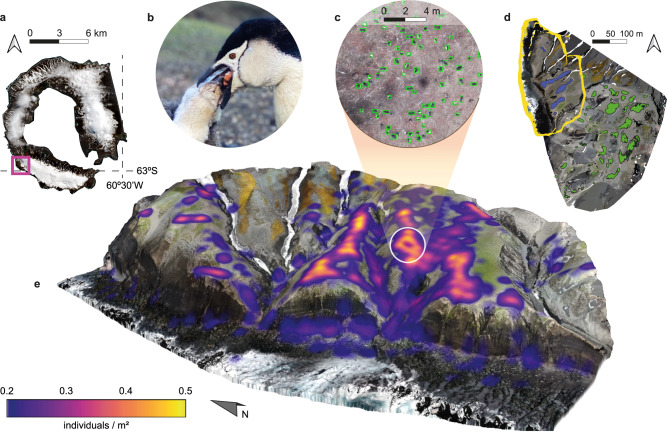
Study site and double approach for iron export estimation. **a** Sentinel-2 10 m resolution image of Deception Island (South Shetland Islands)—Vapour Col (magenta square), where the data for this study were collected, is the second largest Chinstrap penguin rookery on Deception Island, gathering more than 19,000 breeding pairs^65^. **b** Adult individual feeding krill to its chick, their main food source^33,35^, characterized by a bright orange coloration. **c** Chinstrap population census estimated by the deep learning model. Despite certain limitations of the inference over areas with Chinstrap penguin-like artifacts, such as the rocky coastal areas, the model succeeded in accurately detecting the individuals in relevant, high density Chinstrap penguin areas (Supplementary Table 1). **d** Vapour Col photographically available extension. The northern tip of Vapour Col is outlined in yellow and corresponds to the terrain of the panel **e**. Blue and green patches correspond to the output of the non-supervised classification for guano areas^47^. **e** The northern tip of Vapour Col—penguin density is shown as a heatmap, the brightest areas correspond to the breeding zones where the penguins are tightly clustered, matching with the guano accumulation zones.

In this study, we applied a holistic approach that addresses both penguin population dynamics and the amount of Fe exported through their excretion products in relation to penguin biomass, to analyse the Fe input from these seabirds into the marine environment. Using Deception Island’s Vapour Col rookery (Fig. 1a, d, e) as a case study, we focused on one of the most abundant Antarctic penguin species, the Chinstrap penguin (*Pygoscelis antarcticus*) (Fig. 1b). We assessed the Chinstrap penguin relative population status by collecting drone images of the breeding site, data which served as an input for a deep learning model trained to detect penguin individuals (Fig. 1c). With non-supervised classification from drone imagery^47^ (Fig. 1d), we estimated the amount of guano present in the colony, which we cross-validated with the Chinstrap penguin census-based guano accumulation (Fig. 1e). We quantified the Fe contents of guano and calculated the relative guano discharge, based on the Fe concentrations measured in the waters surrounding the rookery and the areas further away. Subsequently, we extrapolated the input of Fe for the global population of the Chinstrap penguin and estimated the magnitude of its contribution to yearly carbon accumulation in the Southern Ocean and nearby regions of the Antarctic Peninsula. These calculations serve as an empirical basis to highlight the role of these penguins as one of the major vertebrate mediators in the pelagic Fe recycling system, prompting a possible ecosystem imbalance caused by their significant population decrease.

## Results

### Water iron enrichment through penguin guano

To evaluate the magnitude of the Chinstrap penguin contribution to the Fe pool in the surrounding waters, we analysed its relative accumulation in the colony and further discharge to the seawater. We examined three guano sample types collected on: ice, soil and from a specific collector designed for sampling uncontaminated soil-free guano (see Methods). Among all guano types, Fe exhibited a mean concentration of 3.0 ± 1.4 mg g^−1^ guano, ranging from 2.3–4.0 mg g^−1^ in the guano collector, with a peak reaching 5.8 mg g^−1^ in the soil guano samples, likely reflecting a certain degree of influence from the soil. These Fe concentrations of guano samples are also consistent with the Chinstrap penguin’s main food source, the Antarctic krill, which acts like long-term Fe reservoir^25,48^.

**Fig. 2:**
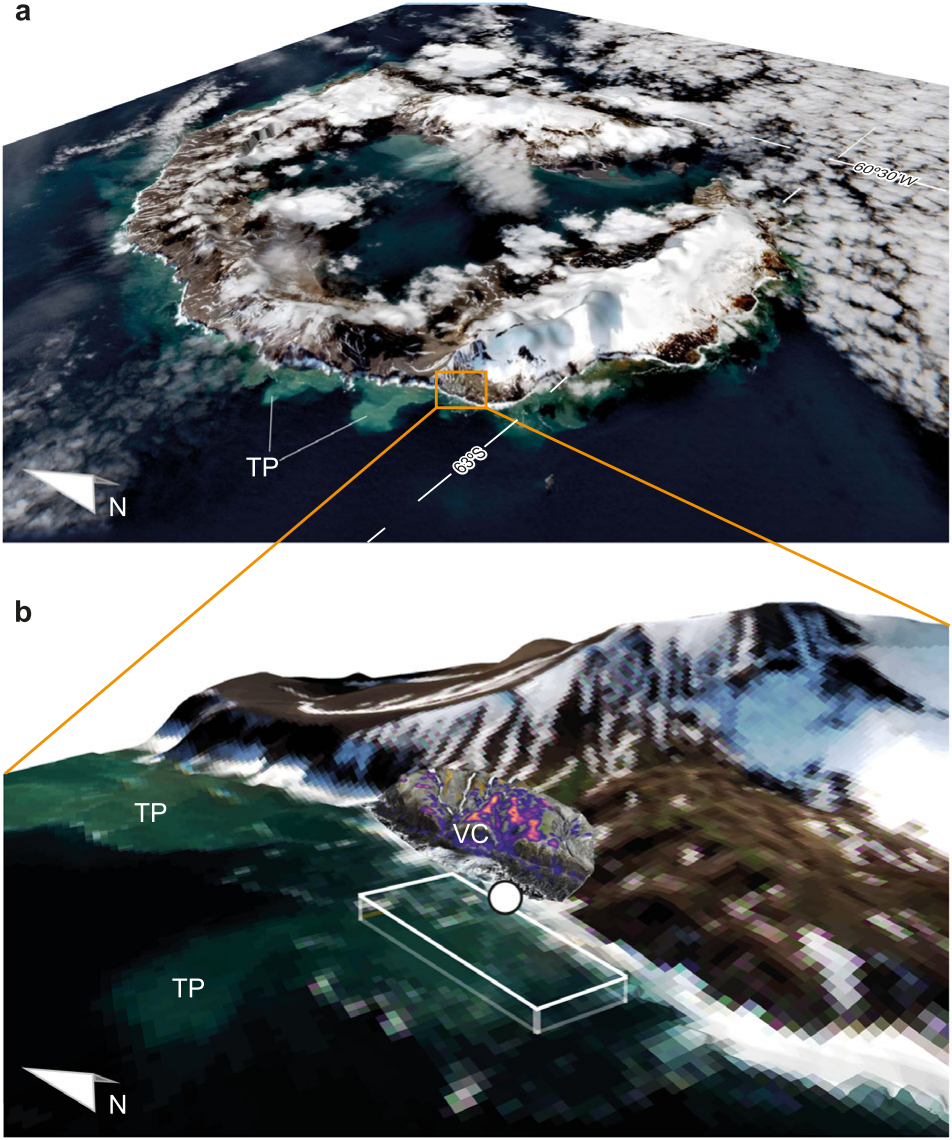
Deception Island coastal high turbidity waters and considered volume for Fe discharge estimation from Vapour Col. **a** Sentinel-2 3-D composition of Deception Island, where areas of high turbidity can be seen flowing westward, next to the Vapour Col colony. **b** Volume section of coastal water under the influence of the penguin colony. The white dot is the surface water sampling location (62°59’29” S, 60°43’32” W) for Fe analysis. To estimate the relative discharge of Fe to the Antarctic waters we consider a calculated seawater volume of 8 × 10^4^ m^3^ with a 2 m average depth near-shore region of 4 × 10^4^ m^2^ (white cuboid). TP Turbidity Plumes originating from the Vapour Col breeding site. VC Vapour Col colony.

To assess the amount of guano excreted in the colony and subsequently obtain its Fe content, we volumetrically characterized, using non-supervised classification^47^, the nesting areas where the majority of the penguins are clustered, i.e., guano-rich zones (GRZs, Fig. 1d, e), resulting in 165.5 tonnes of dry weight guano containing 500 ± 241 kg Fe (see Methods). To cross-check this estimation, we examined an individual-level approximation^42^ using the Chinstrap penguin census as estimated by the deep learning model to produce an output based on the mean excretion rate of each individual per day and its Fe content. We found that according to the 16,725 ± 907 penguins censed by the model, a total of 169.4 t of dry weight guano can be expected in the breeding site considering 120 days as the breeding season duration, containing up to 512 ± 246 kg Fe (see Methods). Subsequently, considering the Fe concentration (0.324 mg L^−1^)^38^ in the coastal water at the specific time of study, we obtained an approximate Fe content of 26 kg in 8 × 10^4^ m^3^ of water under the influence of the Chinstrap penguin turbidity plume (Fig. 2a, b). Therefore, considering the accumulated amount of Fe in the GRZs of the Vapour Col colony using the mean between GRZ volumes and deep learning census, at a given time, 5.2% (3.5–9.7%, combining GRZ volumes and deep learning census uncertainties) of the Fe content is drained into the surrounding waters. These calculations are derived from a specific point in time and location within the colony. To address temporal and spatial variations in the quantity of Fe released and its associated transport efficiency, data from multiple points throughout the breeding season would be required.

### Chinstrap penguin global iron input in the Southern Ocean

Evaluating the Fe contribution of the global Chinstrap penguin population is complex due to its seasonal life cycle, since the individuals leave the colony once the breeding season is over, making it difficult to precisely quantify the guano released into the aquatic environment throughout the year. In this regard, we divided the guano release timing in two periods: breeding and non-breeding seasons. To quantify the fraction of guano that reaches the Antarctic waters during the breeding period, it is necessary to take into account multiple inherent climatic, terrain and biological factors. We consider that guano accumulation and washing responds to a dynamic interaction between factors, including penguin movement, in-situ snow accumulation or melting runoff from surrounding elevated terrain. Therefore, guano can be stored in and released from the colony at any given time, depending on specific environmental conditions, consequently varying the amount of Fe released. Furthermore, terrain geomorphology highly conditions the flow of guano towards the ocean, with higher rates in steep terrains such as Vapour Col. Although we estimated over 5% Fe discharge from the whole colony (see Methods), this fraction can be significantly higher if areas of guano accumulation are located near terrain with sufficient inclination and exposed to meteorological events. For instance, in colonies where the terrain is similar to the northern tip of the Vapour Col colony, which geomorphology features steep hills and cliffs, if only the guano accumulated in this area is taken into account, the amount of Fe released from this zone is over 20% of the total accumulated Fe in it (see Methods). During the non-breeding season, Pygoscelis species, such as Adélie or Chinstrap penguins, have shown to migrate towards areas with high krill concentrations, spending more than 60% of this time in the water, near the northern edges of the pack ice^49–51^. Consequently, to obtain estimates of guano release during the non-breeding season, we used guano production rates previously calculated for the breeding period, and assumed that the deposited guano on the edges of the ice shelves is almost entirely washed into the ocean during the summer season, when the Antarctic winter ice sheet recedes.

**Fig. 3:**
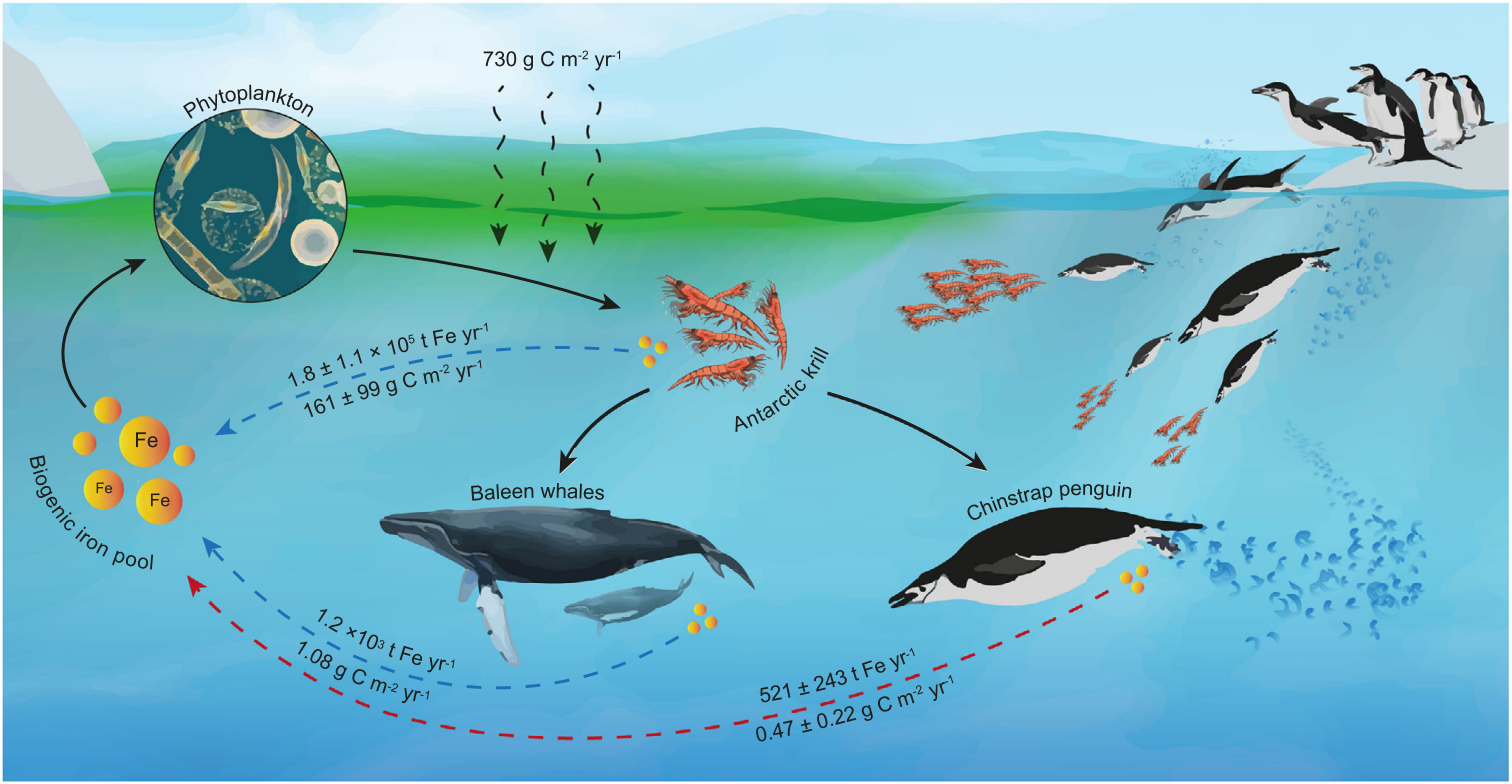
Iron recycling and net primary production stimulation in the Southern Ocean pelagic areas. A perspective of the Chinstrap penguins’ relative Fe input and net primary production stimulation compared to post-whaling Mysticeti^52^ and Antarctic krill^63,66^ Fe fertilizing rates, estimated in this study from a literature model^29^ (see Methods). Carbon assimilation associated with primary production (2 g C m^−*2*^ day^−*1*^, here expressed as annual assimilated C)^67^ refers to the Southern Ocean coastal areas, such as polynyas, marginal ice zones and the continental shelf. Despite the lower penguin Fe contribution compared to that from baleen whales, this number accounts for only one species in contrast to the Mysticeti genus, with Chinstrap numbers being 50% lower than four decades ago^46^, when whales were already subjected to whaling pressure. Solid arrows indicate energy fluxes within the ecosystem. Dashed arrows indicate Fe input and primary production stimulation (from previous studies (blue lines) and the present study (red line)).

Taking into account the described features that influence the release of guano throughout both periods, we considered a conservative 10% average global Fe release efficiency from breeding sites during the reproductive season, corresponding to a reduced fraction (24.5 tonnes Fe yr^−1^) of what is released to the ocean during the non-breeding period. Therefore, considering the current global population of Chinstrap penguin, this species produces up to 521 ± 243 tonnes Fe yr^−1^ (see Methods) (Fig. 3).

**Fig. 4:**
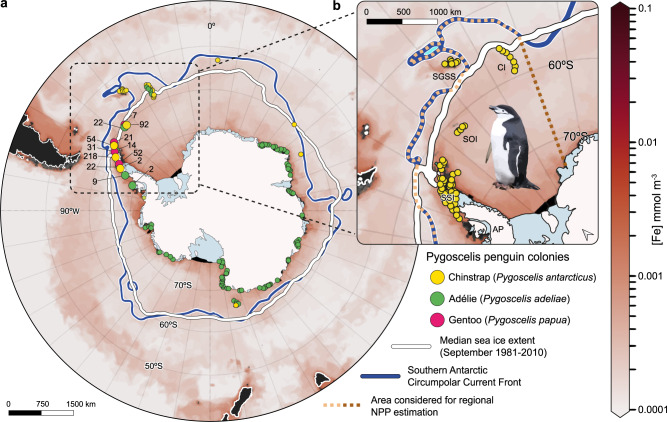
Iron surface concentration in the Southern Ocean and Pygoscelis distribution in the Antarctic continent. **a** Mole concentration of dissolved Fe in sea water in September, 2021, in the Antarctic and Sub-Antarctic regions as provided by the Global Ocean Biogeochemistry Analysis and Forecast product from the Copernicus Marine Service^68^; Line in blue, the Southern Antarctic Circumpolar Front, defined by Fe-depleted areas^2^, bordering the sea ice zones delimited by the white line as the winter median sea ice extent. The Adélie and Gentoo penguin colonies distribution was obtained from the Mapping Application for Penguin Populations and Projected Dynamics^69^. The Chinstrap colonies distribution was obtained from its global population assessment carried out by Strycker et al.^46^. Within the dashed square, a high number of Pygoscelis colonies were clustered, numbers indicate the quantity of packed colonies for each dot. **b** The Chinstrap penguin colonies distribution on the Antarctic Peninsula. The orange dashed line encloses the area considered in this study for the calculation of Net Primary Production (NPP) stimulated regionally by the Chinstrap penguins.

We further explored the yearly net primary productivity (NPP) that is stimulated by Chinstrap penguin Fe input in the Antarctic waters. Using an Fe recycling model proposed for baleen whales^29^, we first calculated the bioavailable Fe, to then estimate total NPP in g C m^−2^ yr^−1^ for the extension of the Southern Ocean (2 × 10^13^ m^2^) and the Antarctic Peninsula waters (4.5 × 10^12^ m^2^, estimated from Fig. 4). We found that considering the 521 tonnes Fe yr^−1^ introduced by the global Chinstrap penguin population, this species can be contributing with 0.12 ± 0.05 to 1.05 ± 0.5 g C m^−2^ yr^−1^, with an estimated mean rate of 0.47 ± 0.22 g C m^−2^ yr^−1^ (see Methods) to the entire Southern Ocean. These values are of the same range of magnitude as the NPP driven by the entire Mysticeti genus’ Fe input^52^, which currently represents an estimated average of 1.08 g C m^−2^ yr^−1^ NPP stimulation, highlighting the significance of the Chinstrap penguin when comparing single- to multi-species Fe export to the Antarctic waters. At regional scale, surrounding the Antarctic Peninsula, Chinstrap populations can stimulate 0.52 ± 0.24 to 4.69 ± 2.19 g C m^−2^ yr^−1^, with a mean of 2.09 ± 1.33 g C m^−2^ yr^−1^, enriching Fe-poor areas of the nearby Antarctic Circumpolar Current (Fig. 4). These calculations, however, should be interpreted as an upper limit of the NPP that the Chinstrap penguins can stimulate, as other limiting factors such as light limitation, nutrient availability or mixing processes are not considered.

## Discussion

Higher Fe concentrations in the Chinstrap penguin guano result especially interesting when compared to other krill-feeding Antarctic species. For example, in baleen whales, who currently recycle up to 1.2 × 10^3^ tonnes Fe yr^−1^ ^52^, Fe content in excrements range between 0.146 ± 0.134 mg g^−1 ^^48^, being at most more than ten times lower than the Fe content in Chinstrap penguin guano found in the present study. The consequent release of this guano to the Southern Ocean represents close to half of the input produced by baleen whales, which highlights the environmental relevance of Chinstrap penguin populations.

Although fluctuating patterns can be noticed between the populations of this seabird, mainly due to the complexity of spatial distribution, an overall decrease in their numbers is evident, especially in the South Shetland Islands, where various studies showed significant drops in the number of individuals^43,44,53,54^. Similar to the rest of the regions inhabited by the Chinstrap penguin, Vapour Col presented a similar negative fluctuation, where a 36% population decrease was reported between 1991 and 2008^45^.

This general negative trend in the Antarctic Peninsula and, by default, in the rest of their breeding sites, can be related to the environmental changes faced by the Southern Ocean region mainly due to climate change effects^55^. Several mechanisms are currently considered as a possible explanation for the decline of Chinstrap penguin populations, arguing possible krill biomass-related causes due to rapid environmental changes in the Southern Ocean. As consequence, a decoupling between krill concentrations and distribution and the nonbreeding winter migrations could be responsible for the decreasing Chinstrap penguin numbers^46^. This downtrend of the Chinstrap penguin population could potentially lead to a similar situation as that experienced by baleen whales, in which their Fe recycling is now up to ten times lower than in the pre-whaling period^48^. In this sense, a global decline of >50% in the Chinstrap penguin numbers has been reported since the 1980s^46^, thus suggesting that only four decades ago, Fe recycling by these seabirds had approximately the same magnitude as that produced by baleen whales subjected to current whaling pressure.

Including the Adélie penguin (*Pygoscelis adeliae*) as the other widely distributed Pygoscelis species (Fig. 4a), with increasing population numbers reaching 10^7^ individuals^56^ with a mainly krill-based diet^34,36^, Pygoscelis Fe input to the Southern Ocean could potentially reach the amount that baleen whales are currently recycling. Therefore, a deeper understanding of the Chinstrap penguin’s (and by extension of the entire Pygoscelis genus) life and prey-consumption cycles, migrations, and breeding-site guano export dynamics, would help improve their conservation status and their impact on Fe recycling in the Antarctic marine ecosystem.

## Methods

### Guano sample collection and chemical analysis

During January and February, 2021, fresh guano samples were collected at the Vapour Col (VC) breeding site. The samples were differentiated according to the collection substrate, since three types of samples were analysed: soil, ice, and guano collected in a “trap”. The traps consist of a polyethylene plastic plate with a PVC frame (40 × 30 cm long), placed in the colony 24 h prior to sampling, to guarantee the collection of fresh samples, avoiding possible contamination from the soil. Fresh guano samples were collected in VC (*n* = 23), and placed manually using a plastic spoon in polyethylene bags or in acid-cleaned vials and stored frozen at −20 °C until analysis. Fe was later extracted with a microwave acid digestion system (MARS-V, CEM) in accordance with the SW-846 EPA Method 3051A^57^. Approximately 0.2 g of guano sample were digested with 10 mL of nitric acid (65%, Suprapur quality) in Teflon vessels, in triplicate. After digestion, Fe contents were analysed (see Fe concentrations in Supplementary Fig. 1 and Supplementary Data 2) using an inductively coupled plasma-mass spectrometry (ICP -MS, iCAP Thermo). Blanks and certified material for digestion and analysis were treated like the samples. The accuracy of the analytical methods was checked using a certified reference material (Lobster hepatopancreas TORT-2), with Fe concentration of 109.0 ± 5.3 μg g^−1^, and a recovery of 103.8 ± 5.0%. The Detection Limit calculated as three times the standard deviation of the blank values was 0.43 μg g^−1^.

### Identification of guano-rich zones

To identify the regions of the colony with the greatest accumulation of guano, i.e., the GRZ, the result of a non-supervised classification^47^ over the VC colony terrain was used (Fig. 1d). Subsequently, the total area of the GRZ predicted by the classification was calculated, representing a time-specific guano layer within VC. The area of these regions served as a baseline for subsequent guano volume and Chinstrap penguin population estimation in VC, as it is considered that the greatest percentage of the Fe export is derived from these zones.

### Chinstrap penguin deep learning-powered census

On February 8, 2021, Deception Island Chinstrap penguin rookeries were photographed during the PiMetAn Project XXXIV Spanish Antarctic campaign^47^ using unmanned aerial vehicles (UAV), commonly known as drones. The photographic dataset used here does not cover the entire extension of VC but only the northern tip of it (NT_VC_) (Supplementary Fig. 2a), and the rest of VC was photographed at a height of 150 m (Supplementary Fig. 2a), thus not suitable for penguin detection due to their small body size. Subsequently, 377 RGB images of NT_VC_ were selected with a resolution of 3000 × 4000 pixels, captured from a flight height of 30 m and speed of 4.9 m s^−1^. The photographic data were obtained using the DJI Mavic 2 Pro UAV equipped with an RGB sensor (Hasselblad Camera) and flights were configured using DJI’s Ground Station Pro photogrammetric flight planning software. The 377 photographs were used to generate an orthomosaic using Agisoft Metashape photogrammetric software.

The model Faster R-CNN^58^ (FRCNN) was used to perform object detection tasks. In the present research FRCNN with ResNet-101 backbone. Training and evaluation tasks were performed using the TensorFlow 2.0 machine learning platform by Google (see training dataset, generated using Roboflow annotation services, in Supplementary Data 1). The model performance was later evaluated by analysing the resulting detection metrics of three regions of interest (ROI) selected within the orthomosaic: ROI 1 and ROI 3, corresponding to a non-coastal area and ROI 2, corresponding to a representative region of a coastal area (Supplementary Fig. 2b, Supplementary Table 1).

The deep learning-powered census set the number of Chinstrap penguins in NT_VC_’s GRZ at 2,265 ± 159. The number of individuals located in outer zones (OZ, i.e., those that are not GRZ) of NT_VC_ was estimated at 1853 ± 130. The previous data were used to calculate the density of penguins for both zones, obtaining 0.52 ± 0.03 ind. m^−2^ for the GRZ and 0.028 ± 0.01 ind. m^−2^ for the OZ. Subsequently, the density extrapolation for the highly overflown (150 m) rest of VC was carried out, using the densities obtained for NT_VC_ and applying them to these new GRZ and OZ. A final number of 7,611 ± 439 individuals were found grouped in GRZs and 4,996 ± 179 in the OZs. Finally, the total number of Chinstrap penguins in the entire VC colony was obtained by adding the output for both zones (Supplementary Table 2).

### Vapour Col Fe accumulation

Two approaches were explored to quantitatively assess the total Fe abundance at a given time in the studied region of the colony (see calculations in Supplementary Table 3). Firstly, the GRZ volumes were calculated to obtain an estimate of the amount of Chinstrap penguin excrements accumulated in the area. Determining an exact thickness of the guano layer for volume calculation was challenging, mainly due to the irregularity of the terrain, varying substrates, accumulation and discharge dynamics, and the scarcity of supporting references for this specific region. Consequently, only a thin layer of 2 cm as a representative thickness of fresh guano accumulation was taken into account. Then, the volume was used to estimate the total Fe content for each of the GRZs, using the following equation:

1 $${g}_{Fe}=w\,Fe\,{a}_{GRZ} \, t\,{d}_{guano}$$

where *g*_*Fe*_ is the total Fe in g, *w* is the dry weight guano fraction, which for seabirds has been estimated at 0.4^59^; *Fe* is the Fe concentration in µg per g of dry guano, *a*_GRZ_ is the area in m^2^ as determined using the non-supervised classification^47^, *t* is the guano layer thickness and *d*_guano_ is the density of the guano obtained in VC (1088.6 kg m^−3^).

To cross-check the output of the Fe content estimation using the GRZ volumes, an individual-level approach presented by Sparaventi et al.^42^ was used according to the following equation:

2 $${g}_{Fe}=Cp\,Fe\,e\,d$$

where *Cp* is the deep learning-based Chinstrap census, *e* is the penguin excretion as g of dry guano per day, estimated at 84.4 g^60^; *d* is the number of days a certain number of penguins remain in the area. As the breeding season begins when the Chinstrap penguins arrive at their colonies between early October and November until, based on census information from the PiMetAn campaign in Vapour Col, the end of February, 120 days were used as the guano production period in the above equation. Comparatively, the total number of Chinstrap penguins present in the colony, and not only in its GRZ, was considered, as it was assumed that each individual must spend time within the nesting areas, thus contributing to guano accumulation.

### Fe export dynamics from Vapour Col colony

During the campaign, one sampling was performed in the VC colony^38^, in a shore area in the vicinity of various depressions between hills which act like “funnels”, both for penguins to go to/from the nests, and for guano runoff to the ocean. The sampling location was used as a starting point from which the volume of water influenced by the guano release was calculated. To determine the boundaries of the water volume (white cuboid, Fig. 2b), two main limiting factors were considered: for the height, the apparent bottom proximity; and for the length and width, the extension of the turbidity plume as viewed from Sentinel-2 satellite. Regarding the depth, there is no data in the sampling point. Hence, relying on the visual observations of the sampling site depth observed from UAV camera^38^, a conservative 2 m of average depth was used. For the length and width of the cuboid, the extension of the turbidity plume extracted from Sentinel-2 satellite RGB image (Fig. 2a, b) was considered. Although similar plumes can be observed emanating from nearby locations, those plumes come from the sediments and snow melting, as there are no penguin colonies located in those spots. Plumes being released from Vapour Col, carry as well the guano runoff, which contains the Fe that was later used to calculate the export efficiency. This guano runoff occurs through the mentioned depressions in the steep terrain, that act like a funnel, as the major part of the VC region is characterized by abrupt cliffs. Therefore, considering this funnel effect and that the observed plume is emanating from the sampling region, the length of the cuboid was extended to cover the visible turbidity plume along the coast. Then, based in the depth assumptions made above regarding the depth, the cuboid was extended 40 m from the coast, for a total of 8 × 10^4^ m^3^ of water. In this study, the dimensions taken into account to consider a certain water volume followed conservative constraints to avoid overestimation of the Fe export, as possible variance introduced in the considered water volume can affect the release efficiency from the breeding site.

Finally, to obtain Fe release efficiencies from the breeding site, the percentage that the Fe in the considered water volume represents from the total accumulated Fe in the guano from ground was calculated. For the whole extension of Vapour Col, 380.2 m^3^ of guano and for the northern tip of the colony, 87.4 m^3^. These guano volumes were used to obtain Fe content and subsequently its percentage being released in the water (see calculations in Supplementary Table 3). Penguin populations and its subsequent produced guano discharge to the water due to climatological and geomorphological features vary during the breeding season, and therefore the guano accumulation and release efficiencies are subjected to fluctuations. In this sense, future research should address this seasonal populational variations within the colony and establish the relation between the guano content and the water Fe concentration to obtain accurate breeding site Fe release efficiencies throughout the entire breeding season.

### Yearly Fe release by the global Chinstrap penguin population

To calculate the yearly amount of Fe released by the Chinstrap penguins to the Southern Ocean waters, two periods were differentiated within the year based on the Fe export efficiency. Firstly, for the breeding season, following the observed trend during late February in Vapour Col, 120 days (November–February) are considered, when the Fe release from the breeding sites is calculated to be a conservative 10% of all produced Fe during this period. For the remaining 245 days of the non-breeding season, given the Chinstrap penguin phenology, and the Antarctic sea ice dynamics, the totality of the produced guano, and subsequently the Fe, is considered to be released into the Southern Ocean for calculations (see calculations in Supplementary Table 4).

### Primary production from iron fertilization

The NPP stimulated by Chinstrap penguin Fe input into the Southern Ocean (PP_Cp_) was calculated using a model for Fe cycling proposed by Ratnarajah et al.^29^ (see calculations in Supplementary Table 5). According to Shatova et al.^40^, it is possible to assume that the major part of the Fe contained in seabird guano will be bioavailable for phytoplankton growth, due to its low pH and organic matter content, which increases solubility. Also, similarly to whales, as discussed by Ratnarajah et al. in their model, penguins swim in the first 100 m of the ocean upper layer, thus depositing Fe-rich guano in the euphotic zone. However, guano that enters in the surrounding waters is not always fully available for the phytoplankton. Guano can be subjected to sinking from the euphotic zone before being assimilated by phytoplankton due to its particulate nature or also be absorbed by bacteria. Considering also the scarcity in literature addressing Fe bioavailability in seabird guano Fe, and mainly observing positive effects on its growth when guano is added to water^40^, to obtain the amount of Fe suitable for phytoplankton usage (*P*_*Cp*_), Fe retention in the photic zone (*p*_Cp_) and the Fe bioavailable for phytoplankton (*ε*_*Cp*_) varied between a wide range of assumed fractions 0.25 (min.), 0.5 (base) and 0.75 (max.):

3 $${P}_{{Cp}}=\frac{{R}_{{Fe}}{p}_{{Cp}}{\varepsilon }_{{Cp}}}{{M}_{{Fe}}}$$

where *R*_*Fe*_ is the Fe mass being released with penguin guano and *M*_*Fe*_ is the Fe molecular mass (55.845 g mol^−1^). Subsequently, considering a 3 µmol Fe: mol C phytoplankton ratio (*u*)^61,62^, an estimate of NPP was obtained for the extension of the Southern Ocean (South of 60°S) and for the waters surrounding the Antarctic Peninsula as follows:

4 $${{PP}}_{{Cp}}=\frac{{P}_{{Cp}}{M}_{C}u}{a}$$

where *M*_*C*_ is the carbon molecular mass (12.01 g mol^−1^). In the same way, to obtain a comparative perspective, NPP was calculated for the current Fe input estimation for baleen whales^52^ and krill^63^.

### Reporting summary

Further information on research design is available in the Nature Portfolio Reporting Summary linked to this article.

## Supplementary information

Supplementary Information

Description of Additional Supplementary Files

Supplementary Data 1

Supplementary Data 2

Reporting Summary

## Data Availability

The training dataset and iron concentrations data generated during the current study are provided in the Supplementary Materials. The training dataset is also available at the Australian Antarctic Data Centre on https://data.aad.gov.au/metadata/PENGUIN_DET_VAPCOL.

## Code availability

The code used in the present study is available on https://github.com/obkorolev/penguin_iron_paper.^64^
