# Supplementary material for: The contribution of penguin guano to the Southern Ocean iron pool
Source: Nat Commun. 2023 Apr 11;14:1781. doi: 10.1038/s41467-023-37132-5 (PMC10090129; doi:10.1038/s41467-023-37132-5)
Supplement: Supplementary file 1 — Supplementary Information [file 41467_2023_37132_MOESM1_ESM.pdf]

The contribution of penguin guano to the Southern Ocean iron pool

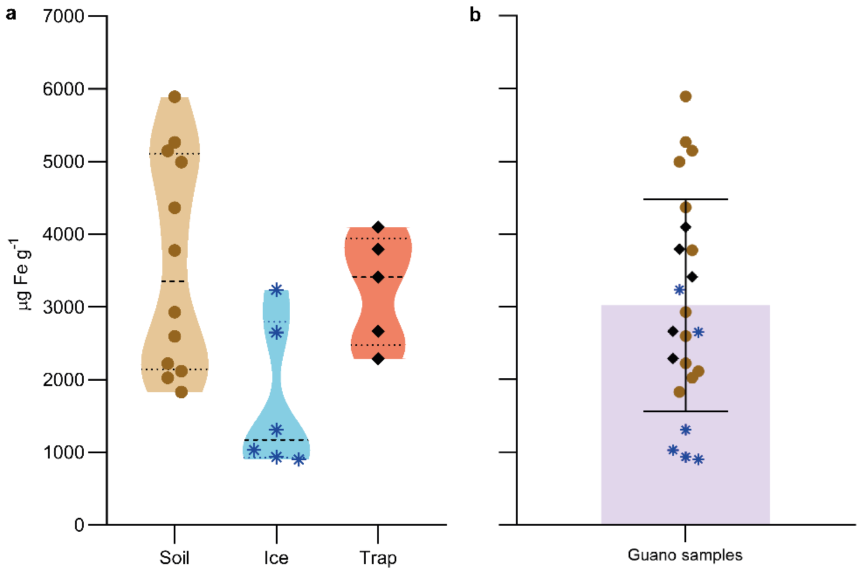

Supplementary Figure 1 | Iron concentration in fresh guano samples collected at Vapour Col. Twelve samples from 10 to 500 g directly from the soil, six samples from 35 to 450 g from the ice and five samples from 13 to 50 g from the guano collectors or "traps". **a).** Soil, ice and trap Fe concentrations. Displaying median, Q1 and Q3, 25th and 75th percentile. **b).** Mean and standard deviation of n=23 guano samples, 3.0 ± 1.4 mg g^{-1} guano.

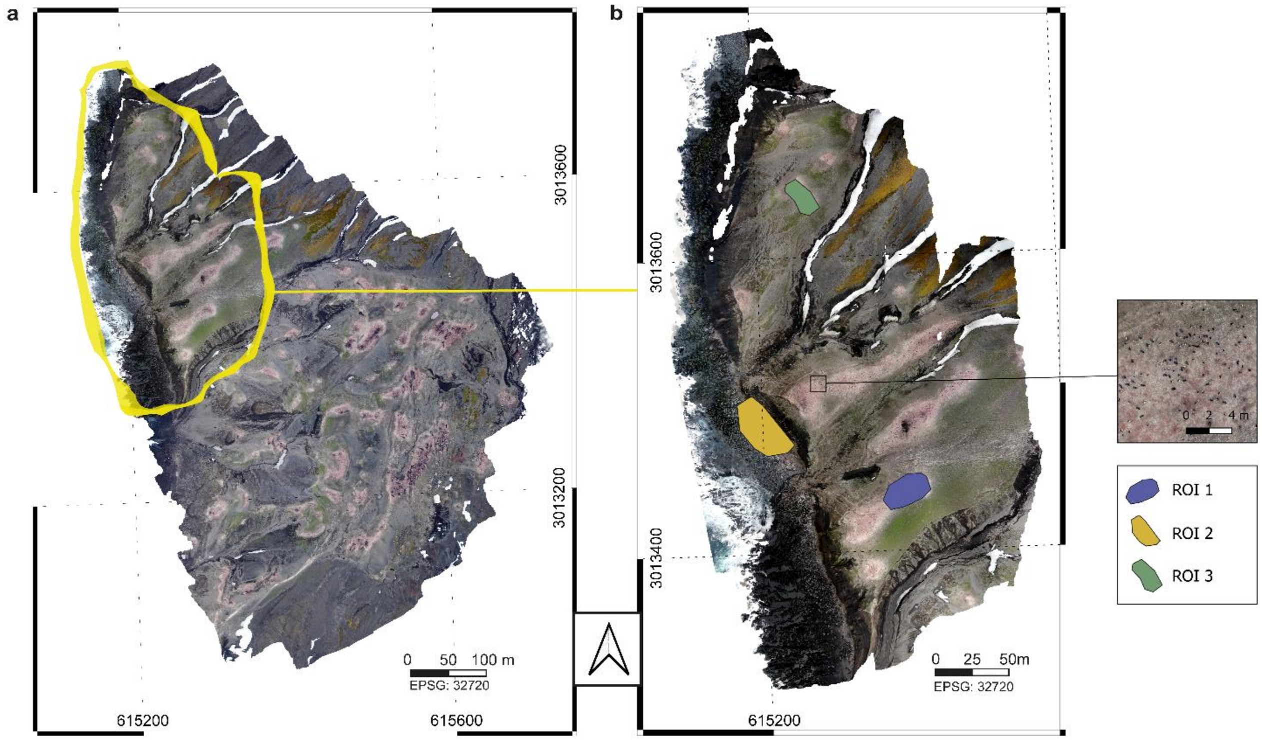

Supplementary Figure 2 | Photographic available data of the Vapour Col colony and Regions of Interest for deep learning model evaluation. a). Vapour Col breeding site. Outlined in yellow, is the northern tip of Vapour Col, NT_{VC}, which was captured 30 m above the ground, yielding an optimum quality for penguin detection. The rest of the colony outside NT_{VC} was captured at a height of 150 m. **b).** Close-up look of NT_{VC}, where the ROIs were selected for deep learning model evaluation. ROI 1 and 3 correspond to a plain, artifact-free area, where the detection had optimum results. ROI 2 corresponds to a rocky coast, with many similar features that compromised the performance of the model. However, this coastal area did not account for a large number of Chinstrap individuals in a visual examination of the terrain in the orthomosaic.

Supplementary Table 1 | Detection metrics yielded by the deep-learning model for the Chinstrap census. For ROI 1 and 2, the ability of the model to find the maximum number of penguin individuals (recall) and the confidence that they were actually penguins (precision) is very high (max = 1), mainly due to the relatively plain terrain and scarcity of penguin-like objects. ROI 2 showed good precision but a low recall, due to a relatively high confidence threshold (0.6) and the presence of rocks and water foam.

	Equation	ROI 1	ROI 2	ROI 3
Precision (P)	true positives / (true positives + false positives)	0.98	0.9	0.97
Recall (R)	true positives / (true positives + false negatives)	0.89	0.31	0.92
F1 score	2 × P × R / (P + R)	0.93	0.47	0.94

Supplementary Table 2 | Detected Chinstrap penguins, areas and penguin densities. To calculate their abundance in the highly overflown area (150 m) density extrapolation was performed for the GRZ and for the outer guano-free zones (OZ).

	GRZ (m^2)	OZ (m^2)	Individuals in GRZ	Individuals in OZ	Density GRZ (ind. m^{-2})	Density OZ (ind. m^{-2})	Total individuals
NT_{VC}	4,370	66,216	2,265±159	1,853±130	0.52±0.03	0.028±0.01	4,118±289
Rest of Vapour Col overflown at 150 m	14,636	178,400	7,611±439	4,996±179	0.52±0.03	0.028±0.01	12,607±618
							16,725±907

Supplementary Table 3 | Calculations of the relative Fe content in Vapour Col. Two methods were proposed to cross-validate the amount of Fe present in the colony at a certain time. The first accounts for Fe obtained using guano-rich zone volumes from non-supervised classification[1], and the second uses the estimated excretion per penguin individual, whose total number is obtained from the deep-learning census of the present study. NTVC: calculations for the northern tip of Vapour Col. WHC: Calculations for the whole colony. Referenced parameters[2,3].

<table>
<tr><th>Parameter</th><th>Reference</th><th>Value</th><th>Equation</th><th>Result</th><th>Uncertainty</th></tr>
<tr><td colspan="6">Fe calculation based on non-supervised guano rich zone classification</td></tr>
<tr><td>w</td><td>[2]</td><td>0.4</td><td rowspan="3">kg Fe NTVC = 0.4 × 3,02 × 4,370 × 1.0886 × 0.02</td><td rowspan="3">115 kg Fe</td><td rowspan="3">62 - 168 kg Fe</td></tr>
<tr><td>Fe</td><td>Present study</td><td>3.02 mg g^{-1}</td></tr>
<tr><td rowspan="2">a_{GRZ}</td><td rowspan="2">Present study</td><td>4,370 m^2 (NTCV)</td></tr>
<tr><td>19,006 m^2 (WHC)</td><td rowspan="3">kg Fe WHC = 0.4 × 3,02 × 19,006 × 1.0886 × 0.02</td><td rowspan="3">500 kg Fe</td><td rowspan="3">268 – 732 kg Fe</td></tr>
<tr><td>d_{guano}</td><td>Present study</td><td>1.0886 × 10^6 g m^{-3}</td></tr>
<tr><td>t</td><td>Assumed, present study</td><td>0.02 m</td></tr>
<tr><td colspan="6">Fe calculation based on deep learning Chinstrap penguin census</td></tr>
<tr><td rowspan="2">Chinstrap penguin</td><td rowspan="2">Present study</td><td>4,118 (NTCV)</td><td rowspan="3">kg Fe NTVC = (4,118 × 3,02 × 84.4 × 120)</td><td rowspan="3">126 kg Fe</td><td rowspan="3">117 – 135 kg Fe</td></tr>
<tr><td>16,725 (WHC)</td></tr>
<tr><td>Fe</td><td>Present study</td><td>3.02 mg g^{-1}</td></tr>
<tr><td>e</td><td>[3]</td><td>84.4 g</td><td>kg Fe WHC = (16,725 × 3,02 × 84.4 × 120)</td><td>512 kg Fe</td><td>266 – 758 kg Fe</td></tr>
<tr><td>d</td><td>Assumed, present study</td><td>120 days</td><td></td><td></td><td></td></tr>
</table>

Supplementary Table 4 | Calculations of the yearly global Fe release by the Chinstrap penguin population. Two periods within the Chinstrap penguin life cycle were differentiated, associating respective guano export efficiencies. From the results of this study, it is estimated that 10% of the guano accumulated in the breeding sites is exported to the Southern Ocean waters, while its entirety should be released during the foraging season. Referenced parameters[3,4]

Parameter	Reference	Value	Equation	Result	Total export	Uncertainty
Breeding season						
Fe	Present study	$3\ mg\ g^{-1}$				
Total individuals	[4]	8×10^6				
e	[3]	84.4 g	$t\ Fe = 3 \times 8 \times 10^6 \times 84.4 \times 120 \times 0.1$	24.3 t Fe		
d	Assumed, present study	120 days				
Fe release efficiency	Present study	0.1				
Non-breeding season					$521\ t\ Fe\ yr^{-1}$	278 – 764 t Fe
Fe	Present study	$3\ mg\ g^{-1}$				
Total individuals	[4]	8×10^6				
e	[3]	84.4 g	$t\ Fe = 3 \times 8 \times 10^6 \times 84.4 \times 245 \times 1$	496.3 t Fe		
d	Assumed, present study	245 days				
Fe release efficiency	Present study	1				

Supplementary Table 5 | Calculations for net primary production stimulated by Chinstrap penguin Fe input. In the same way as calculated here for the Chinstrap penguin, NPP was also obtained for krill, based on ref.[5,6] and for baleen whales, based on ref.[7]. Referenced parameters[7–10]. * Refers to the selected parameter of retained Fe in the photic zone and the bioavailable Fe for the phytoplankton, as the model proposed by Ratnarajah et al.[8] assumed values of 0.25 and 0.75 as max. and min.

Step / Parameter	Reference	Value	Equation	Result	Uncertainty
Chinstrap penguin annual Fe input in the Southern Ocean	Present study	521 tonnes Fe yr^{-1}			278 – 764 tonnes Fe yr^{-1}
Fraction of Fe retained in the photic zone	8	0.25/0.5*/0.75	5.21×10^{8} g Fe yr^{-1} × 0.5	2.61×10^{8} g Fe yr^{-1} retained in the photic zone	1.39 – 3.82 $\times10^{8}$ g Fe yr^{-1}
Fraction of Fe bioavailable for phytoplankton	8	0.25/0.5*/0.75	2.61×10^{8} g Fe yr^{-1} × 0.5	1.31 $\times10^{8}$ g Fe yr^{-1} bioavailable for phyto.	0.7 – 1.91 $\times10^{8}$ g Fe yr^{-1}
Fe : C ratio of phytoplankton in the Southern Ocean	7,9,10	3 µmol Fe : mol C			1 – 6 µmol Fe : mol C
Fe molecular weight		55.845 g mol^{-1}			
g of carbon incorporated into phytoplankton biomass (NPP)	Present study		(1.31 $\times10^{8}$ g Fe yr^{-1} × 0.018 mol Fe × 10^{6} µmol Fe × mol C × 12.01 g C) / (g Fe × mol Fe × 3 µmol Fe × mol C)	9.44 × 10^{12} g C yr^{-1} incorporated into phyto.	5.04 – 13.76 × 10^{12} g C yr^{-1}
Southern Ocean area (South of 60ºS)		2 × 10^{13} m^{2}			
Rate of NPP stimulation by Chinstrap penguin guano input	Present study		(9.44 × 10^{12} g C yr^{-1} incorporated into phyto.) / (2 × 10^{13} m^{2})	0.47 g C m^{-2} yr^{-1} incorporated into phyto.	0.25 – 0.69 g C m^{-2} yr^{-1}

References

1. Tovar-Sánchez, A., Román, A., Roque-Atienza, D. & Navarro, G. Applications of unmanned aerial vehicles in Antarctic environmental research. *Sci Rep* **11**, 21717 (2021).
2. De La Peña-Lastra, S. Seabird droppings: Effects on a global and local level. *Science of The Total Environment* **754**, 142148 (2021).
3. Sun, L. & Xie, Z. Relics: Penguin Population Programs. *Science Progress* **84**, 31–44 (2001).
4. BirdLife International (2022) Species factsheet: Pygoscelis antarcticus. *BirdLife International* http://www.birdlife.org/ (2022).
5. Maldonado, M. T., Surma, S. & Pakhomov, E. A. Southern Ocean biological iron cycling in the pre-whaling and present ecosystems. *Phil. Trans. R. Soc. A.* **374**, 20150292 (2016).
6. Böckmann, S. *et al.* Salp fecal pellets release more bioavailable iron to Southern Ocean phytoplankton than krill fecal pellets. *Current Biology* **31**, 2737-2746.e3 (2021).
7. Savoca, M. S. *et al.* Baleen whale prey consumption based on high-resolution foraging measurements. *Nature* **599**, 85–90 (2021).
8. Ratnarajah, L. *et al.* A preliminary model of iron fertilisation by baleen whales and Antarctic krill in the Southern Ocean: Sensitivity of primary productivity estimates to parameter uncertainty. *Ecological Modelling* **320**, 203–212 (2016).
9. Strzepek, R. F., Maldonado, M. T., Hunter, K. A., Frew, R. D. & Boyd, P. W. Adaptive strategies by Southern Ocean phytoplankton to lessen iron limitation: Uptake of organically complexed iron and reduced cellular iron requirements. *Limnol. Oceanogr.* **56**, 1983–2002 (2011).
10. Twining, B. S., Baines, S. B. & Fisher, N. S. Element stoichiometries of individual plankton cells collected during the Southern Ocean Iron Experiment (SOFeX). *Limnol. Oceanogr.* **49**, 2115–2128 (2004).
