# Supplementary material for: The contribution of penguin guano to the Southern Ocean iron pool
Source: Nat Commun. 2023 Apr 11;14:1781. doi: 10.1038/s41467-023-37132-5 (PMC10090129; doi:10.1038/s41467-023-37132-5)
Supplement: Supplementary file 2 — Description of Additional Supplementary Files [file 41467_2023_37132_MOESM2_ESM.pdf]

Description of Additional Supplementary Files

File Name: Supplementary Data 1
Description: Zip file containing the dataset used to train and test object detection model for penguin detection in Vapour Col.

File Name: Supplementary Data 2
Description: Excel sheet containing the iron concentration found in all collected samples (n=23) and its respective substrate, penguin species, and location.
