# Supplementary figures and images for: The contribution of penguin guano to the Southern Ocean iron pool (part 2 of 2)
Source: Nat Commun. 2023 Apr 11;14:1781. doi: 10.1038/s41467-023-37132-5 (PMC10090129; doi:10.1038/s41467-023-37132-5)

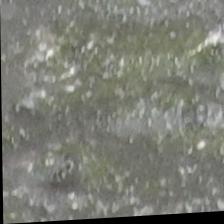

Supplement: Supplementary file 3 — Supplementary Data 1 [file 41467_2023_37132_MOESM3_ESM.zip › train/test_11_1_jpg.rf.5de81c588214a5ba468f8ca64c51a318.jpg]

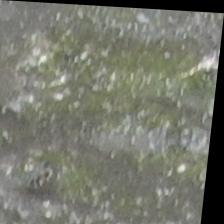

Supplement: Supplementary file 3 — Supplementary Data 1 [file 41467_2023_37132_MOESM3_ESM.zip › train/test_11_1_jpg.rf.e695d823d4d117c1d9004438fdbaec7e.jpg]

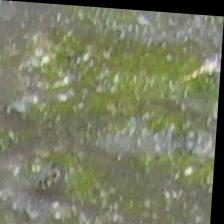

Supplement: Supplementary file 3 — Supplementary Data 1 [file 41467_2023_37132_MOESM3_ESM.zip › train/test_11_1_jpg.rf.f8993e07caae9c1342979b665cfc4d9d.jpg]

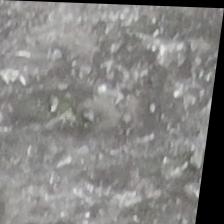

Supplement: Supplementary file 3 — Supplementary Data 1 [file 41467_2023_37132_MOESM3_ESM.zip › train/test_11_3_jpg.rf.09f2e35d69ebb94b3dfd05990ed35427.jpg]

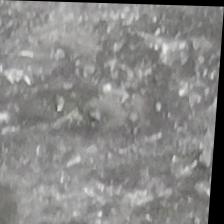

Supplement: Supplementary file 3 — Supplementary Data 1 [file 41467_2023_37132_MOESM3_ESM.zip › train/test_11_3_jpg.rf.2d8412a33629675dcace2b20df7df4b6.jpg]

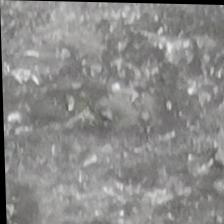

Supplement: Supplementary file 3 — Supplementary Data 1 [file 41467_2023_37132_MOESM3_ESM.zip › train/test_11_3_jpg.rf.fa5066f1d800ce6c254d231166d8ac42.jpg]

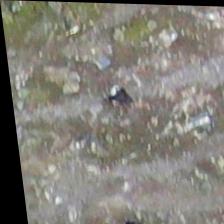

Supplement: Supplementary file 3 — Supplementary Data 1 [file 41467_2023_37132_MOESM3_ESM.zip › train/test_11_5_jpg.rf.0990e599e4e76ba3e797f5e4da1b2055.jpg]

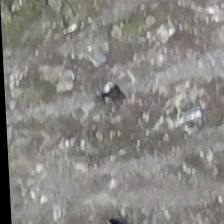

Supplement: Supplementary file 3 — Supplementary Data 1 [file 41467_2023_37132_MOESM3_ESM.zip › train/test_11_5_jpg.rf.667f2be822f998d483789c252f502635.jpg]

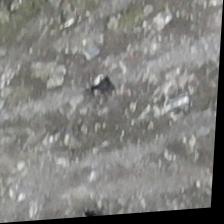

Supplement: Supplementary file 3 — Supplementary Data 1 [file 41467_2023_37132_MOESM3_ESM.zip › train/test_11_5_jpg.rf.c894760f51df518c568194f0265af375.jpg]

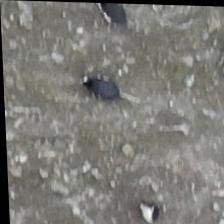

Supplement: Supplementary file 3 — Supplementary Data 1 [file 41467_2023_37132_MOESM3_ESM.zip › train/test_11_6_jpg.rf.808d4cc256922c137f070e3f0a6ec5fd.jpg]

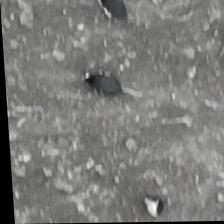

Supplement: Supplementary file 3 — Supplementary Data 1 [file 41467_2023_37132_MOESM3_ESM.zip › train/test_11_6_jpg.rf.c8e8dbb152c3f0fb3c0e597c9cdea0b3.jpg]

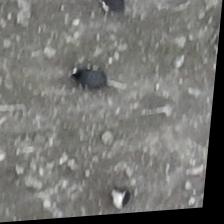

Supplement: Supplementary file 3 — Supplementary Data 1 [file 41467_2023_37132_MOESM3_ESM.zip › train/test_11_6_jpg.rf.ff84b1561d6f238813b1a5265c2f3ab4.jpg]

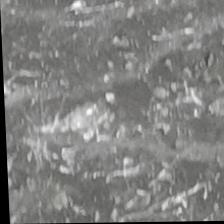

Supplement: Supplementary file 3 — Supplementary Data 1 [file 41467_2023_37132_MOESM3_ESM.zip › train/test_11_7_jpg.rf.20657eac92983389af3098bcc1b50dec.jpg]

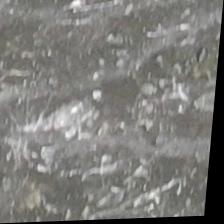

Supplement: Supplementary file 3 — Supplementary Data 1 [file 41467_2023_37132_MOESM3_ESM.zip › train/test_11_7_jpg.rf.58763eb9e4957a00432509e111c420b3.jpg]

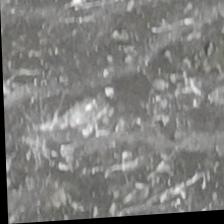

Supplement: Supplementary file 3 — Supplementary Data 1 [file 41467_2023_37132_MOESM3_ESM.zip › train/test_11_7_jpg.rf.65caf85be106bd0a5a3207e592b7cbff.jpg]

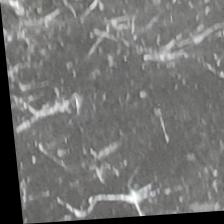

Supplement: Supplementary file 3 — Supplementary Data 1 [file 41467_2023_37132_MOESM3_ESM.zip › train/test_11_9_jpg.rf.0c3bef58bc0806f8c53eef56b3ac5d1d.jpg]

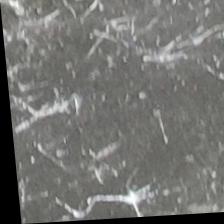

Supplement: Supplementary file 3 — Supplementary Data 1 [file 41467_2023_37132_MOESM3_ESM.zip › train/test_11_9_jpg.rf.c03bbc8edd47425d2c250d75e2083cb7.jpg]

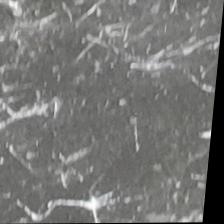

Supplement: Supplementary file 3 — Supplementary Data 1 [file 41467_2023_37132_MOESM3_ESM.zip › train/test_11_9_jpg.rf.c7f37b2923e2f8031c8e75cfa5ecb973.jpg]

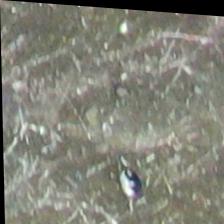

Supplement: Supplementary file 3 — Supplementary Data 1 [file 41467_2023_37132_MOESM3_ESM.zip › train/test_12_10_jpg.rf.1deb4e3b425cf0cf029bb305757efe23.jpg]

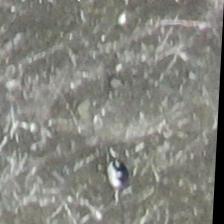

Supplement: Supplementary file 3 — Supplementary Data 1 [file 41467_2023_37132_MOESM3_ESM.zip › train/test_12_10_jpg.rf.6b4fc54a4101d7170a7e38fa69cbcbdf.jpg]

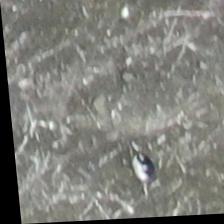

Supplement: Supplementary file 3 — Supplementary Data 1 [file 41467_2023_37132_MOESM3_ESM.zip › train/test_12_10_jpg.rf.8e80b42f6a9242d324fdab10372db5de.jpg]

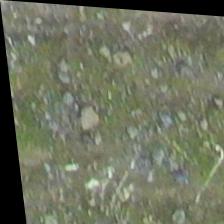

Supplement: Supplementary file 3 — Supplementary Data 1 [file 41467_2023_37132_MOESM3_ESM.zip › train/test_12_12_jpg.rf.19121839a8ead68d709a0513bc6ffbb5.jpg]

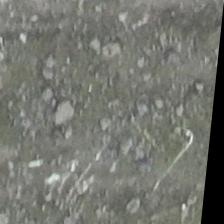

Supplement: Supplementary file 3 — Supplementary Data 1 [file 41467_2023_37132_MOESM3_ESM.zip › train/test_12_12_jpg.rf.7cdb7ae0ca989e1827ad6bc9830eb5bd.jpg]

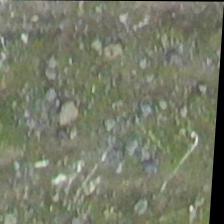

Supplement: Supplementary file 3 — Supplementary Data 1 [file 41467_2023_37132_MOESM3_ESM.zip › train/test_12_12_jpg.rf.d0f762716b350406ea64c4d8401683f0.jpg]

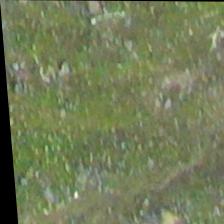

Supplement: Supplementary file 3 — Supplementary Data 1 [file 41467_2023_37132_MOESM3_ESM.zip › train/test_12_13_jpg.rf.6dfe6d39618901cb622e08355e8ed4e0.jpg]

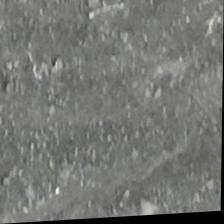

Supplement: Supplementary file 3 — Supplementary Data 1 [file 41467_2023_37132_MOESM3_ESM.zip › train/test_12_13_jpg.rf.83018304376087b10272618e70502bf7.jpg]

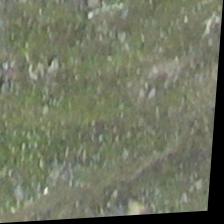

Supplement: Supplementary file 3 — Supplementary Data 1 [file 41467_2023_37132_MOESM3_ESM.zip › train/test_12_13_jpg.rf.c0fe9d128b302742fb26bc329ef76a2d.jpg]

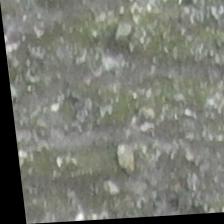

Supplement: Supplementary file 3 — Supplementary Data 1 [file 41467_2023_37132_MOESM3_ESM.zip › train/test_12_1_jpg.rf.a8cec8a908ed5a2eea9558efbb518f66.jpg]

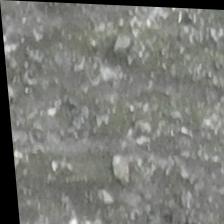

Supplement: Supplementary file 3 — Supplementary Data 1 [file 41467_2023_37132_MOESM3_ESM.zip › train/test_12_1_jpg.rf.c694bdac46133d5819704db5829e5dfd.jpg]

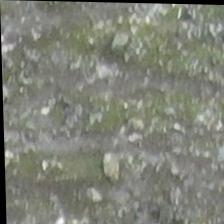

Supplement: Supplementary file 3 — Supplementary Data 1 [file 41467_2023_37132_MOESM3_ESM.zip › train/test_12_1_jpg.rf.d68bde88e2667cd094a9f05024135a79.jpg]

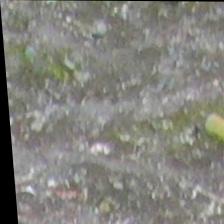

Supplement: Supplementary file 3 — Supplementary Data 1 [file 41467_2023_37132_MOESM3_ESM.zip › train/test_12_2_jpg.rf.469453875216ee3d6a490a4c0bc3393c.jpg]

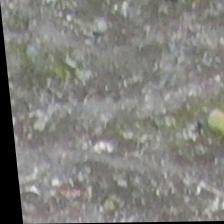

Supplement: Supplementary file 3 — Supplementary Data 1 [file 41467_2023_37132_MOESM3_ESM.zip › train/test_12_2_jpg.rf.d225419fa14f94d4a3a098c2331d79cc.jpg]

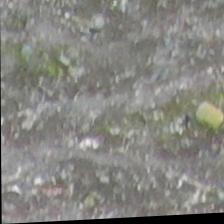

Supplement: Supplementary file 3 — Supplementary Data 1 [file 41467_2023_37132_MOESM3_ESM.zip › train/test_12_2_jpg.rf.dc3aa2e25ec651a46e3ba1da45bd03b0.jpg]

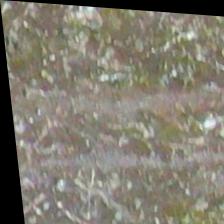

Supplement: Supplementary file 3 — Supplementary Data 1 [file 41467_2023_37132_MOESM3_ESM.zip › train/test_12_6_jpg.rf.2cd87f2f0ae2ceeb6d59093dd38506f4.jpg]

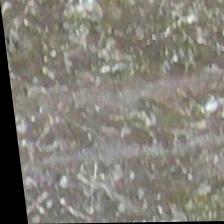

Supplement: Supplementary file 3 — Supplementary Data 1 [file 41467_2023_37132_MOESM3_ESM.zip › train/test_12_6_jpg.rf.87a2d5cf91ee3e398321f6d36a5f5243.jpg]

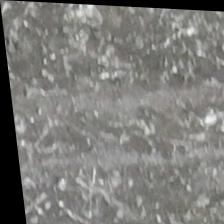

Supplement: Supplementary file 3 — Supplementary Data 1 [file 41467_2023_37132_MOESM3_ESM.zip › train/test_12_6_jpg.rf.97260bbed42050da4019b096b7a681e1.jpg]

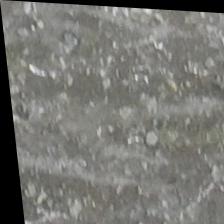

Supplement: Supplementary file 3 — Supplementary Data 1 [file 41467_2023_37132_MOESM3_ESM.zip › train/test_12_7_jpg.rf.061566cb385dd468949e264aefc1a8e9.jpg]

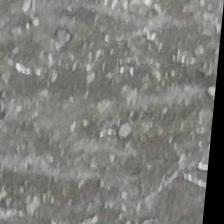

Supplement: Supplementary file 3 — Supplementary Data 1 [file 41467_2023_37132_MOESM3_ESM.zip › train/test_12_7_jpg.rf.13a84038b5776bc9dbf6917a194eee33.jpg]

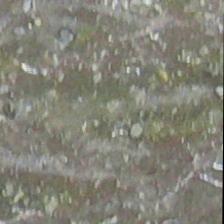

Supplement: Supplementary file 3 — Supplementary Data 1 [file 41467_2023_37132_MOESM3_ESM.zip › train/test_12_7_jpg.rf.b8ced60c95c2272607f6049a41663ce0.jpg]

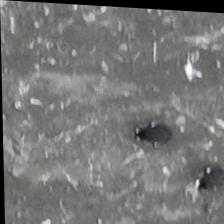

Supplement: Supplementary file 3 — Supplementary Data 1 [file 41467_2023_37132_MOESM3_ESM.zip › train/test_12_8_jpg.rf.087c1a3d93e460065ce61f3a26969068.jpg]

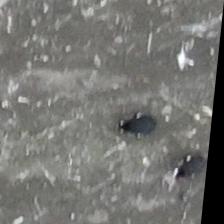

Supplement: Supplementary file 3 — Supplementary Data 1 [file 41467_2023_37132_MOESM3_ESM.zip › train/test_12_8_jpg.rf.0c0e6e4e933b4036b96d3ea1041a323e.jpg]

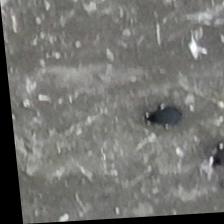

Supplement: Supplementary file 3 — Supplementary Data 1 [file 41467_2023_37132_MOESM3_ESM.zip › train/test_12_8_jpg.rf.db09f862db1808fc324ae2ad4eee1d96.jpg]

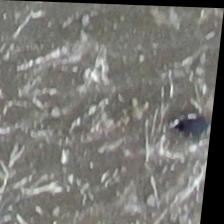

Supplement: Supplementary file 3 — Supplementary Data 1 [file 41467_2023_37132_MOESM3_ESM.zip › train/test_12_9_jpg.rf.1eb1bba2b9d84763c9b59aaaf2a7abea.jpg]

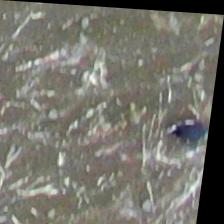

Supplement: Supplementary file 3 — Supplementary Data 1 [file 41467_2023_37132_MOESM3_ESM.zip › train/test_12_9_jpg.rf.44ed3353a72e3997c6a88b3af581c3b4.jpg]

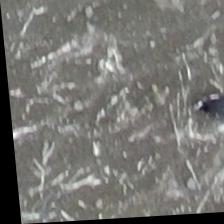

Supplement: Supplementary file 3 — Supplementary Data 1 [file 41467_2023_37132_MOESM3_ESM.zip › train/test_12_9_jpg.rf.c605c54960ef6bd9a0ea86ff5509ac4d.jpg]

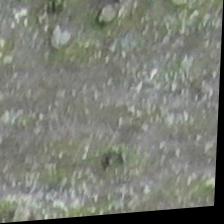

Supplement: Supplementary file 3 — Supplementary Data 1 [file 41467_2023_37132_MOESM3_ESM.zip › train/test_13_0_jpg.rf.3a1c28cb069a2e08aa7f0dccf25e2fa0.jpg]

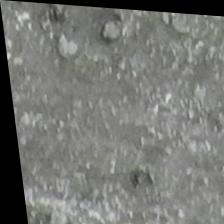

Supplement: Supplementary file 3 — Supplementary Data 1 [file 41467_2023_37132_MOESM3_ESM.zip › train/test_13_0_jpg.rf.554d81d05d57524455a8f305e09e1653.jpg]

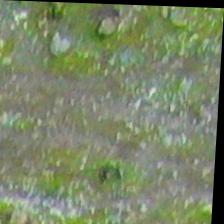

Supplement: Supplementary file 3 — Supplementary Data 1 [file 41467_2023_37132_MOESM3_ESM.zip › train/test_13_0_jpg.rf.fcfcb37f1557da385aaadcb0817fcb15.jpg]

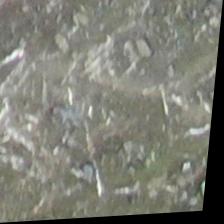

Supplement: Supplementary file 3 — Supplementary Data 1 [file 41467_2023_37132_MOESM3_ESM.zip › train/test_13_10_jpg.rf.357cb13bb61d99c120a73eead6f85fba.jpg]

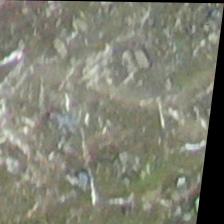

Supplement: Supplementary file 3 — Supplementary Data 1 [file 41467_2023_37132_MOESM3_ESM.zip › train/test_13_10_jpg.rf.dd683fcb829668bb60586162994fda24.jpg]

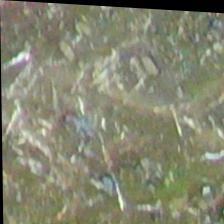

Supplement: Supplementary file 3 — Supplementary Data 1 [file 41467_2023_37132_MOESM3_ESM.zip › train/test_13_10_jpg.rf.edcf178b4dd5dca2ab36a5d29f60f964.jpg]

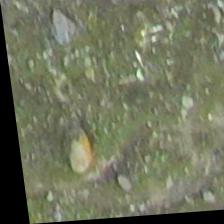

Supplement: Supplementary file 3 — Supplementary Data 1 [file 41467_2023_37132_MOESM3_ESM.zip › train/test_13_12_jpg.rf.5b33eaf5e27bb7b094bafcbddd998b76.jpg]

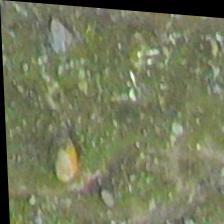

Supplement: Supplementary file 3 — Supplementary Data 1 [file 41467_2023_37132_MOESM3_ESM.zip › train/test_13_12_jpg.rf.5da0450d06b1ca0312b3f2a3be9f515d.jpg]

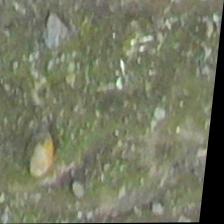

Supplement: Supplementary file 3 — Supplementary Data 1 [file 41467_2023_37132_MOESM3_ESM.zip › train/test_13_12_jpg.rf.e7407f2d1cc78bf4ef001b7a4932d422.jpg]

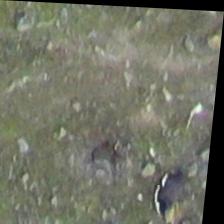

Supplement: Supplementary file 3 — Supplementary Data 1 [file 41467_2023_37132_MOESM3_ESM.zip › train/test_13_13_jpg.rf.5e40db809690b941a5d3e871dc641997.jpg]

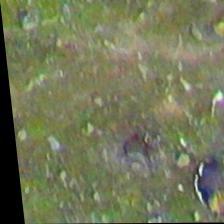

Supplement: Supplementary file 3 — Supplementary Data 1 [file 41467_2023_37132_MOESM3_ESM.zip › train/test_13_13_jpg.rf.74b258b72915084a854ef1f489ce316e.jpg]

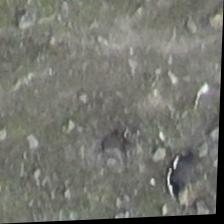

Supplement: Supplementary file 3 — Supplementary Data 1 [file 41467_2023_37132_MOESM3_ESM.zip › train/test_13_13_jpg.rf.eb1694aba66b031da33a7be6bbdef2f7.jpg]

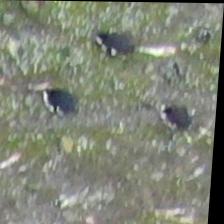

Supplement: Supplementary file 3 — Supplementary Data 1 [file 41467_2023_37132_MOESM3_ESM.zip › train/test_13_1_jpg.rf.44da8b6014dfed1b62ae1b3c4b9ec4d7.jpg]

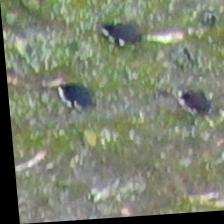

Supplement: Supplementary file 3 — Supplementary Data 1 [file 41467_2023_37132_MOESM3_ESM.zip › train/test_13_1_jpg.rf.48044186504e58f35a9ed6625c6f8ea3.jpg]

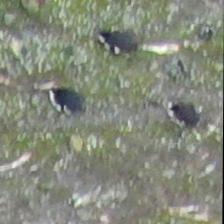

Supplement: Supplementary file 3 — Supplementary Data 1 [file 41467_2023_37132_MOESM3_ESM.zip › train/test_13_1_jpg.rf.fd6daee4d5e7fb9131566500159fa06f.jpg]

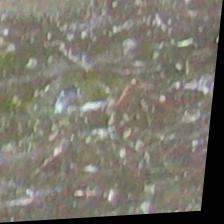

Supplement: Supplementary file 3 — Supplementary Data 1 [file 41467_2023_37132_MOESM3_ESM.zip › train/test_13_7_jpg.rf.12956b6777bfde05795954c37ebdc3ca.jpg]

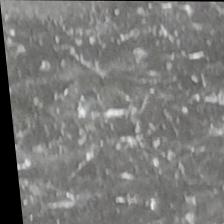

Supplement: Supplementary file 3 — Supplementary Data 1 [file 41467_2023_37132_MOESM3_ESM.zip › train/test_13_7_jpg.rf.16b8be3d79f0bde0c6c3ba6a77063d6f.jpg]

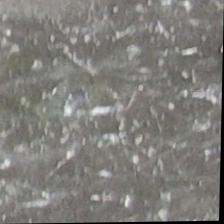

Supplement: Supplementary file 3 — Supplementary Data 1 [file 41467_2023_37132_MOESM3_ESM.zip › train/test_13_7_jpg.rf.1f8d0d1ece55cf9faf8bd8fae041541e.jpg]

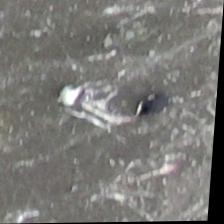

Supplement: Supplementary file 3 — Supplementary Data 1 [file 41467_2023_37132_MOESM3_ESM.zip › train/test_13_8_jpg.rf.195a48bbe9941eb60f5b961ea7079d6a.jpg]

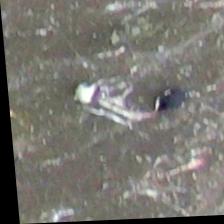

Supplement: Supplementary file 3 — Supplementary Data 1 [file 41467_2023_37132_MOESM3_ESM.zip › train/test_13_8_jpg.rf.4ebf8d92f8353e5663a3b4f086504bd6.jpg]

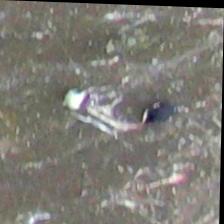

Supplement: Supplementary file 3 — Supplementary Data 1 [file 41467_2023_37132_MOESM3_ESM.zip › train/test_13_8_jpg.rf.65930e2e5c6688ff004617d1cd36029b.jpg]

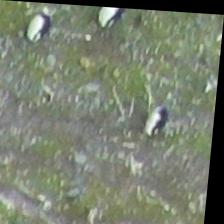

Supplement: Supplementary file 3 — Supplementary Data 1 [file 41467_2023_37132_MOESM3_ESM.zip › train/test_14_0_jpg.rf.3eb2bdab7398af734245b69a2b023c33.jpg]

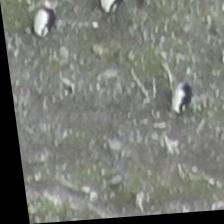

Supplement: Supplementary file 3 — Supplementary Data 1 [file 41467_2023_37132_MOESM3_ESM.zip › train/test_14_0_jpg.rf.9e72fd65d6a9471b681d68877c657116.jpg]

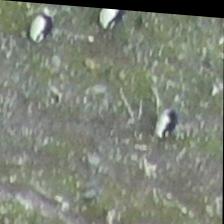

Supplement: Supplementary file 3 — Supplementary Data 1 [file 41467_2023_37132_MOESM3_ESM.zip › train/test_14_0_jpg.rf.fd8178f6eb1b5188ec0e271fb6132aa5.jpg]

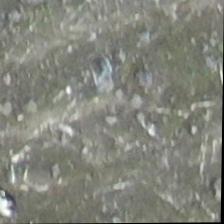

Supplement: Supplementary file 3 — Supplementary Data 1 [file 41467_2023_37132_MOESM3_ESM.zip › train/test_14_10_jpg.rf.0e31d79f2795a65356bd2b726d2d75cd.jpg]

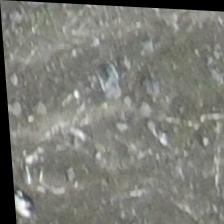

Supplement: Supplementary file 3 — Supplementary Data 1 [file 41467_2023_37132_MOESM3_ESM.zip › train/test_14_10_jpg.rf.7e5f0fb2eed0545011b9f00cfa53e1ba.jpg]

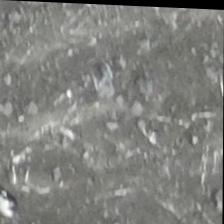

Supplement: Supplementary file 3 — Supplementary Data 1 [file 41467_2023_37132_MOESM3_ESM.zip › train/test_14_10_jpg.rf.a6a1af37fa1560b0f941228152596d51.jpg]

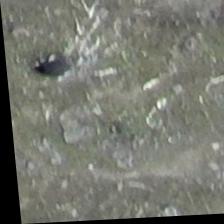

Supplement: Supplementary file 3 — Supplementary Data 1 [file 41467_2023_37132_MOESM3_ESM.zip › train/test_14_12_jpg.rf.18181c011f75a7a856a90d1199b7c29b.jpg]

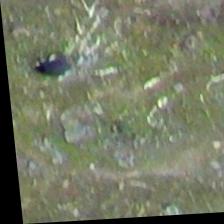

Supplement: Supplementary file 3 — Supplementary Data 1 [file 41467_2023_37132_MOESM3_ESM.zip › train/test_14_12_jpg.rf.95bf5c83aae2da7dc4c1eb81f426915f.jpg]

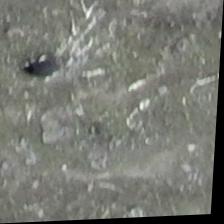

Supplement: Supplementary file 3 — Supplementary Data 1 [file 41467_2023_37132_MOESM3_ESM.zip › train/test_14_12_jpg.rf.fdd9ae8a42a2ddbb0d68c96d7676c603.jpg]

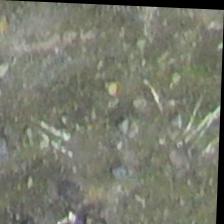

Supplement: Supplementary file 3 — Supplementary Data 1 [file 41467_2023_37132_MOESM3_ESM.zip › train/test_14_13_jpg.rf.06edc07c97f36f32cfd3475fc51a2b27.jpg]

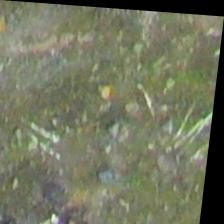

Supplement: Supplementary file 3 — Supplementary Data 1 [file 41467_2023_37132_MOESM3_ESM.zip › train/test_14_13_jpg.rf.52145d6475be21a2c811a5c07e76674c.jpg]

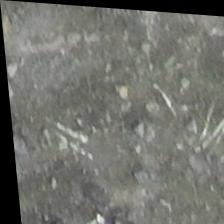

Supplement: Supplementary file 3 — Supplementary Data 1 [file 41467_2023_37132_MOESM3_ESM.zip › train/test_14_13_jpg.rf.9f3f79ade525bd7fd293a87a6ac1180a.jpg]

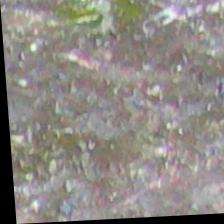

Supplement: Supplementary file 3 — Supplementary Data 1 [file 41467_2023_37132_MOESM3_ESM.zip › train/test_14_3_jpg.rf.264379cab58e2b0e1df518de34042a4d.jpg]

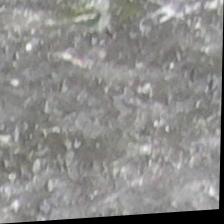

Supplement: Supplementary file 3 — Supplementary Data 1 [file 41467_2023_37132_MOESM3_ESM.zip › train/test_14_3_jpg.rf.b16e7858425aa52b7f15168accfa0c67.jpg]

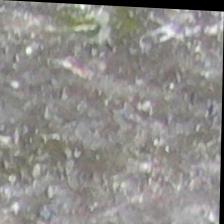

Supplement: Supplementary file 3 — Supplementary Data 1 [file 41467_2023_37132_MOESM3_ESM.zip › train/test_14_3_jpg.rf.b77fbfba6cbbc3563c71258f8110b5ea.jpg]

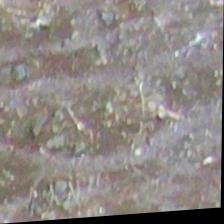

Supplement: Supplementary file 3 — Supplementary Data 1 [file 41467_2023_37132_MOESM3_ESM.zip › train/test_14_4_jpg.rf.5f97beb3715d5e9444f9fc5887eca288.jpg]

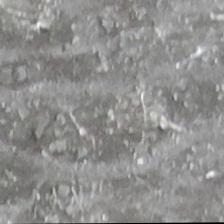

Supplement: Supplementary file 3 — Supplementary Data 1 [file 41467_2023_37132_MOESM3_ESM.zip › train/test_14_4_jpg.rf.60a3703e0e008b3e04f11b04f2042856.jpg]

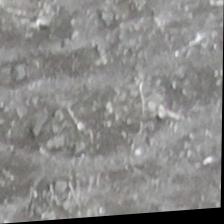

Supplement: Supplementary file 3 — Supplementary Data 1 [file 41467_2023_37132_MOESM3_ESM.zip › train/test_14_4_jpg.rf.f469600a4f19561793b207ff93cfdd38.jpg]

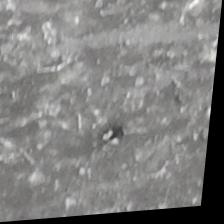

Supplement: Supplementary file 3 — Supplementary Data 1 [file 41467_2023_37132_MOESM3_ESM.zip › train/test_14_5_jpg.rf.37fd4a29337b6ed375c0dbcb5c306c0d.jpg]

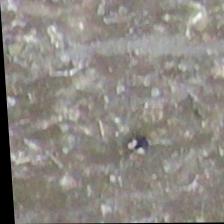

Supplement: Supplementary file 3 — Supplementary Data 1 [file 41467_2023_37132_MOESM3_ESM.zip › train/test_14_5_jpg.rf.4c6de661345dfadbad34d28fd41f755d.jpg]

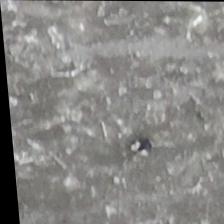

Supplement: Supplementary file 3 — Supplementary Data 1 [file 41467_2023_37132_MOESM3_ESM.zip › train/test_14_5_jpg.rf.73d7d4e8444b15771d4262ebbd88a488.jpg]

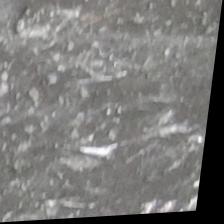

Supplement: Supplementary file 3 — Supplementary Data 1 [file 41467_2023_37132_MOESM3_ESM.zip › train/test_14_6_jpg.rf.0926a7a5b42f4de62c34a27f6f774563.jpg]

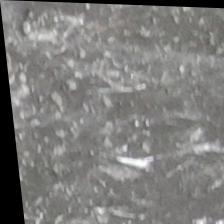

Supplement: Supplementary file 3 — Supplementary Data 1 [file 41467_2023_37132_MOESM3_ESM.zip › train/test_14_6_jpg.rf.780ae00b58bf97176f53a695e6e662ec.jpg]

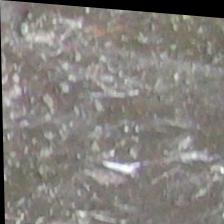

Supplement: Supplementary file 3 — Supplementary Data 1 [file 41467_2023_37132_MOESM3_ESM.zip › train/test_14_6_jpg.rf.98495c6204c8744c21cdc7d7908d1b58.jpg]

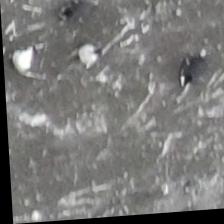

Supplement: Supplementary file 3 — Supplementary Data 1 [file 41467_2023_37132_MOESM3_ESM.zip › train/test_14_7_jpg.rf.06faa4cda67fdd7d74d956a558af7807.jpg]

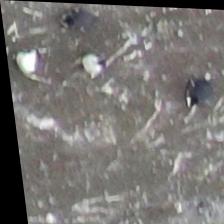

Supplement: Supplementary file 3 — Supplementary Data 1 [file 41467_2023_37132_MOESM3_ESM.zip › train/test_14_7_jpg.rf.6323cb25d371b738f43c3dfcb754f6bc.jpg]

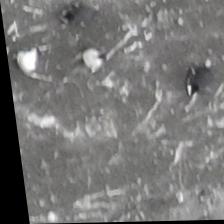

Supplement: Supplementary file 3 — Supplementary Data 1 [file 41467_2023_37132_MOESM3_ESM.zip › train/test_14_7_jpg.rf.c994494acc224690ceb0a7f4d8399ae9.jpg]

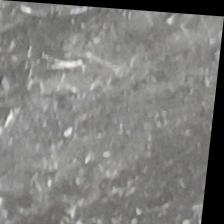

Supplement: Supplementary file 3 — Supplementary Data 1 [file 41467_2023_37132_MOESM3_ESM.zip › train/test_14_8_jpg.rf.a184e4773fe008ba45cced92a7785d58.jpg]

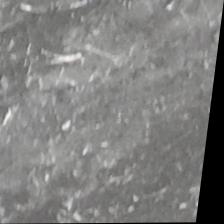

Supplement: Supplementary file 3 — Supplementary Data 1 [file 41467_2023_37132_MOESM3_ESM.zip › train/test_14_8_jpg.rf.b07d5ef956c72ef64efa75d4a1906781.jpg]

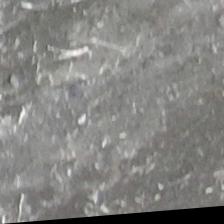

Supplement: Supplementary file 3 — Supplementary Data 1 [file 41467_2023_37132_MOESM3_ESM.zip › train/test_14_8_jpg.rf.b523c9441d186eac6bf5923586de60bc.jpg]

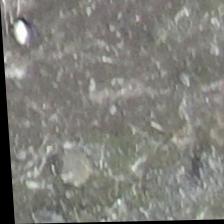

Supplement: Supplementary file 3 — Supplementary Data 1 [file 41467_2023_37132_MOESM3_ESM.zip › train/test_14_9_jpg.rf.40a5f930b60be083b37bad73c1cfb4a6.jpg]
