# Supplementary figures and images for: The contribution of penguin guano to the Southern Ocean iron pool (part 1 of 2)
Source: Nat Commun. 2023 Apr 11;14:1781. doi: 10.1038/s41467-023-37132-5 (PMC10090129; doi:10.1038/s41467-023-37132-5)

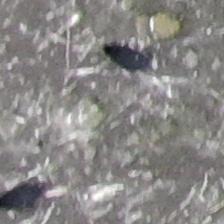

Supplement: Supplementary file 3 — Supplementary Data 1 [file 41467_2023_37132_MOESM3_ESM.zip › test/test_0_10_jpg.rf.03c0a6988581f92b16615f1dcaea6217.jpg]

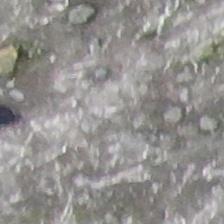

Supplement: Supplementary file 3 — Supplementary Data 1 [file 41467_2023_37132_MOESM3_ESM.zip › test/test_0_4_jpg.rf.0c177351196bab218134675f69a081b1.jpg]

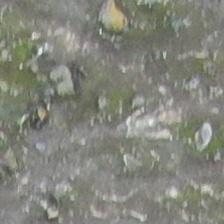

Supplement: Supplementary file 3 — Supplementary Data 1 [file 41467_2023_37132_MOESM3_ESM.zip › test/test_10_1_jpg.rf.a203fa6e68907f3aa8a829f1881fa839.jpg]

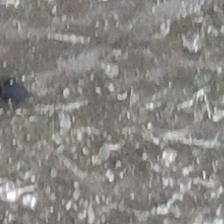

Supplement: Supplementary file 3 — Supplementary Data 1 [file 41467_2023_37132_MOESM3_ESM.zip › test/test_10_6_jpg.rf.2980d32edc550ea3ca6073ad62b4ef58.jpg]

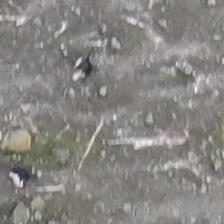

Supplement: Supplementary file 3 — Supplementary Data 1 [file 41467_2023_37132_MOESM3_ESM.zip › test/test_11_4_jpg.rf.f9b9edc8d2b90b106194a7faecb63613.jpg]

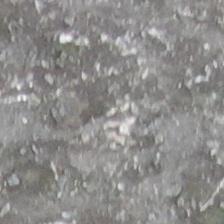

Supplement: Supplementary file 3 — Supplementary Data 1 [file 41467_2023_37132_MOESM3_ESM.zip › test/test_12_3_jpg.rf.ed6efc8abe36fd1b47353a8a5a0fc438.jpg]

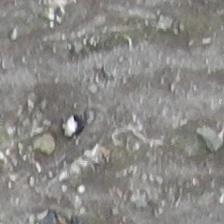

Supplement: Supplementary file 3 — Supplementary Data 1 [file 41467_2023_37132_MOESM3_ESM.zip › test/test_12_4_jpg.rf.a6949c4654c62cafb546e9d37cdba67d.jpg]

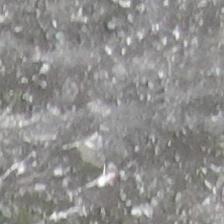

Supplement: Supplementary file 3 — Supplementary Data 1 [file 41467_2023_37132_MOESM3_ESM.zip › test/test_13_3_jpg.rf.6099bc4ceecfbec67c5eb08462bc3bfe.jpg]

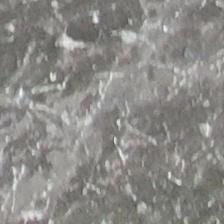

Supplement: Supplementary file 3 — Supplementary Data 1 [file 41467_2023_37132_MOESM3_ESM.zip › test/test_13_9_jpg.rf.ca2e43b348f41f1b24aa1046b2b6e0b0.jpg]

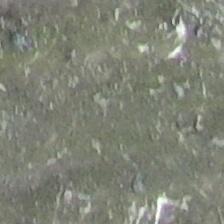

Supplement: Supplementary file 3 — Supplementary Data 1 [file 41467_2023_37132_MOESM3_ESM.zip › test/test_15_12_jpg.rf.9ca9e91730fb28bbb53e2f22174e6c43.jpg]

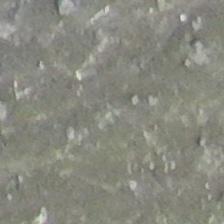

Supplement: Supplementary file 3 — Supplementary Data 1 [file 41467_2023_37132_MOESM3_ESM.zip › test/test_17_11_jpg.rf.8ee859b2ede3d8e656d27007c19f8629.jpg]

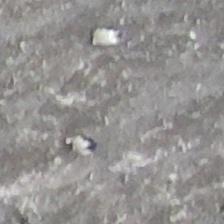

Supplement: Supplementary file 3 — Supplementary Data 1 [file 41467_2023_37132_MOESM3_ESM.zip › test/test_17_6_jpg.rf.aadf84a6f39e602016437ae20c0c03ef.jpg]

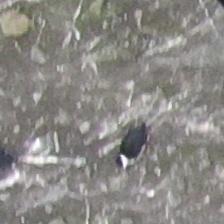

Supplement: Supplementary file 3 — Supplementary Data 1 [file 41467_2023_37132_MOESM3_ESM.zip › test/test_1_5_jpg.rf.ca2b7979c35412d4050b87e904ee1a8f.jpg]

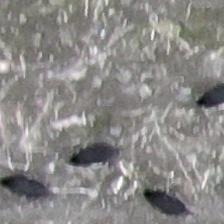

Supplement: Supplementary file 3 — Supplementary Data 1 [file 41467_2023_37132_MOESM3_ESM.zip › test/test_1_6_jpg.rf.832c82620364254037a20217b80488c6.jpg]

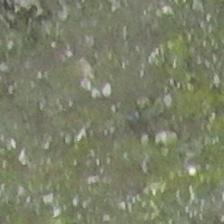

Supplement: Supplementary file 3 — Supplementary Data 1 [file 41467_2023_37132_MOESM3_ESM.zip › test/test_2_0_jpg.rf.7dbf691e7b0adfdfca72cb7011de5dad.jpg]

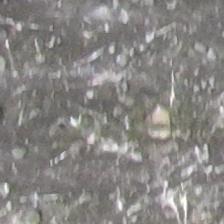

Supplement: Supplementary file 3 — Supplementary Data 1 [file 41467_2023_37132_MOESM3_ESM.zip › test/test_2_8_jpg.rf.3aec29c6c0167048b498a3ba51b4124f.jpg]

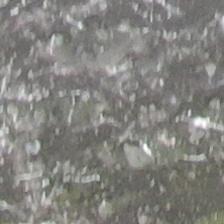

Supplement: Supplementary file 3 — Supplementary Data 1 [file 41467_2023_37132_MOESM3_ESM.zip › test/test_4_11_jpg.rf.6e4ba849a4a520d29fa05a8981b57c02.jpg]

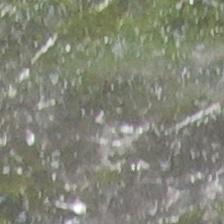

Supplement: Supplementary file 3 — Supplementary Data 1 [file 41467_2023_37132_MOESM3_ESM.zip › test/test_4_2_jpg.rf.149b2fbee5d6c896f2c5b41ff5a533f2.jpg]

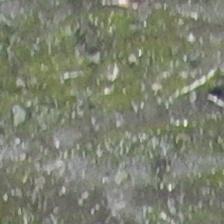

Supplement: Supplementary file 3 — Supplementary Data 1 [file 41467_2023_37132_MOESM3_ESM.zip › test/test_5_1_jpg.rf.497d6d7acdc071d8c69e5ab83030c478.jpg]

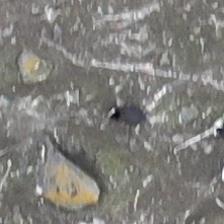

Supplement: Supplementary file 3 — Supplementary Data 1 [file 41467_2023_37132_MOESM3_ESM.zip › test/test_8_7_jpg.rf.f99b7a6ef0ead2b9cb3c5ac630cd5504.jpg]

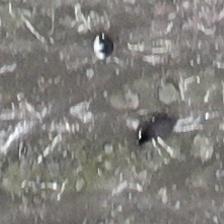

Supplement: Supplementary file 3 — Supplementary Data 1 [file 41467_2023_37132_MOESM3_ESM.zip › test/test_8_9_jpg.rf.af81e849ed64a36126d60b911c0976de.jpg]

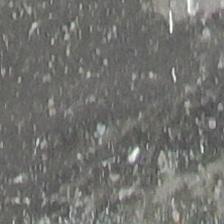

Supplement: Supplementary file 3 — Supplementary Data 1 [file 41467_2023_37132_MOESM3_ESM.zip › test/test_9_12_jpg.rf.e7eb4b1a8621a6afd51ddf1c86c27629.jpg]

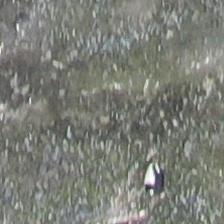

Supplement: Supplementary file 3 — Supplementary Data 1 [file 41467_2023_37132_MOESM3_ESM.zip › test/test_9_13_jpg.rf.695e1b3e9f5b9e998e8552c3ad63ba7f.jpg]

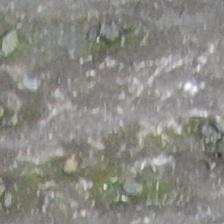

Supplement: Supplementary file 3 — Supplementary Data 1 [file 41467_2023_37132_MOESM3_ESM.zip › test/test_9_2_jpg.rf.7aaf0c92da58425d5e0fd68eb1a26eec.jpg]

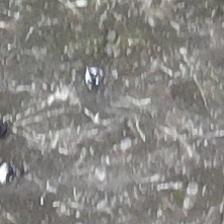

Supplement: Supplementary file 3 — Supplementary Data 1 [file 41467_2023_37132_MOESM3_ESM.zip › test/test_9_7_jpg.rf.429b62e466b70216e8915ecee17f6fad.jpg]

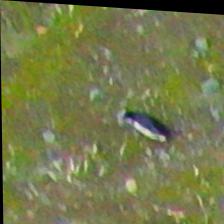

Supplement: Supplementary file 3 — Supplementary Data 1 [file 41467_2023_37132_MOESM3_ESM.zip › train/test_0_0_jpg.rf.62033f4b0dd7cb2316b8be66b3eedeab.jpg]

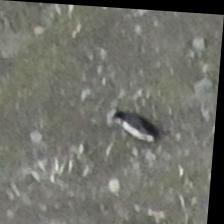

Supplement: Supplementary file 3 — Supplementary Data 1 [file 41467_2023_37132_MOESM3_ESM.zip › train/test_0_0_jpg.rf.c4fd950742d4646e3b1c67b99bda0bb7.jpg]

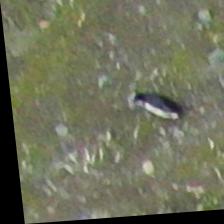

Supplement: Supplementary file 3 — Supplementary Data 1 [file 41467_2023_37132_MOESM3_ESM.zip › train/test_0_0_jpg.rf.e88a58467354e6bd4c4d7eb725683094.jpg]

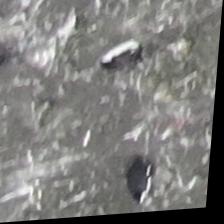

Supplement: Supplementary file 3 — Supplementary Data 1 [file 41467_2023_37132_MOESM3_ESM.zip › train/test_0_11_jpg.rf.5419265b85f95be3984d2c7e2290cece.jpg]

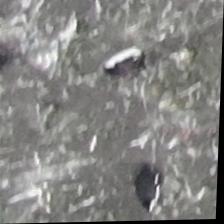

Supplement: Supplementary file 3 — Supplementary Data 1 [file 41467_2023_37132_MOESM3_ESM.zip › train/test_0_11_jpg.rf.5e5e1c0195abd39f888e4535e34bf6cf.jpg]

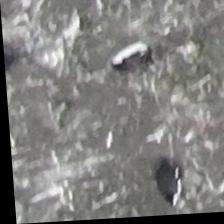

Supplement: Supplementary file 3 — Supplementary Data 1 [file 41467_2023_37132_MOESM3_ESM.zip › train/test_0_11_jpg.rf.89c51a087617245c0f4632aeb1d6d388.jpg]

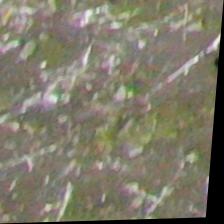

Supplement: Supplementary file 3 — Supplementary Data 1 [file 41467_2023_37132_MOESM3_ESM.zip › train/test_0_12_jpg.rf.a0999f4b1bceffe52e6decc385ecaae6.jpg]

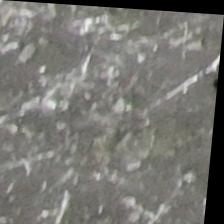

Supplement: Supplementary file 3 — Supplementary Data 1 [file 41467_2023_37132_MOESM3_ESM.zip › train/test_0_12_jpg.rf.b9c26b42430a9fa9e3b1cc75e56761bc.jpg]

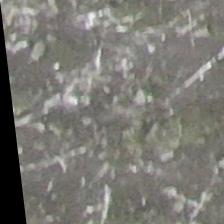

Supplement: Supplementary file 3 — Supplementary Data 1 [file 41467_2023_37132_MOESM3_ESM.zip › train/test_0_12_jpg.rf.cf5561f4a1c81bce27d2d2da7139a062.jpg]

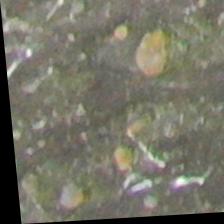

Supplement: Supplementary file 3 — Supplementary Data 1 [file 41467_2023_37132_MOESM3_ESM.zip › train/test_0_13_jpg.rf.0674a1eb0de6a62ad6f5ce9be1070f1b.jpg]

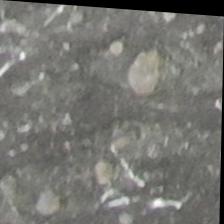

Supplement: Supplementary file 3 — Supplementary Data 1 [file 41467_2023_37132_MOESM3_ESM.zip › train/test_0_13_jpg.rf.63528228f4f5619505a48e92a2d773db.jpg]

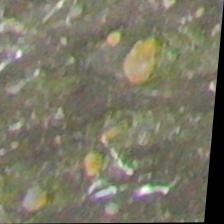

Supplement: Supplementary file 3 — Supplementary Data 1 [file 41467_2023_37132_MOESM3_ESM.zip › train/test_0_13_jpg.rf.912dd196b56a93886aa54b97597f010d.jpg]

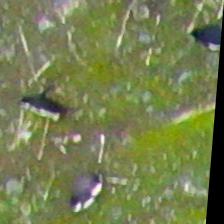

Supplement: Supplementary file 3 — Supplementary Data 1 [file 41467_2023_37132_MOESM3_ESM.zip › train/test_0_1_jpg.rf.74e3940c1b6227889b42fd4341b441db.jpg]

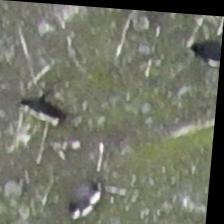

Supplement: Supplementary file 3 — Supplementary Data 1 [file 41467_2023_37132_MOESM3_ESM.zip › train/test_0_1_jpg.rf.9461b0d3a82d63bc42c3b4d7848f7f95.jpg]

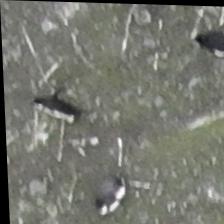

Supplement: Supplementary file 3 — Supplementary Data 1 [file 41467_2023_37132_MOESM3_ESM.zip › train/test_0_1_jpg.rf.d57fd839df01d3c0fbe0b4117b60e648.jpg]

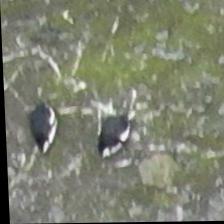

Supplement: Supplementary file 3 — Supplementary Data 1 [file 41467_2023_37132_MOESM3_ESM.zip › train/test_0_2_jpg.rf.0604ab03e22172aa1a8259d2a9d41050.jpg]

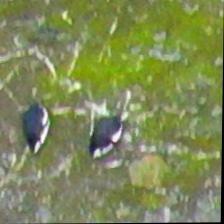

Supplement: Supplementary file 3 — Supplementary Data 1 [file 41467_2023_37132_MOESM3_ESM.zip › train/test_0_2_jpg.rf.5428837a5deb42cd4b39076b83ac0d74.jpg]

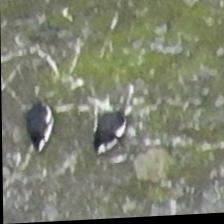

Supplement: Supplementary file 3 — Supplementary Data 1 [file 41467_2023_37132_MOESM3_ESM.zip › train/test_0_2_jpg.rf.99223857f19a73b68c905a3579d32a10.jpg]

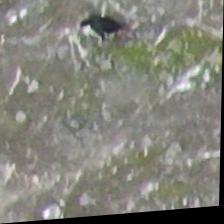

Supplement: Supplementary file 3 — Supplementary Data 1 [file 41467_2023_37132_MOESM3_ESM.zip › train/test_0_3_jpg.rf.8c3ae6ac80f71206b96061d7ec2a654a.jpg]

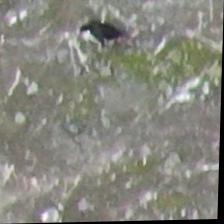

Supplement: Supplementary file 3 — Supplementary Data 1 [file 41467_2023_37132_MOESM3_ESM.zip › train/test_0_3_jpg.rf.9e52ca615ed13b2c2373dbc428afda79.jpg]

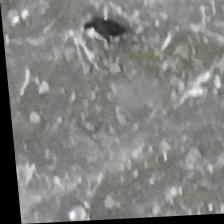

Supplement: Supplementary file 3 — Supplementary Data 1 [file 41467_2023_37132_MOESM3_ESM.zip › train/test_0_3_jpg.rf.e66fe2d20b51883e36270906a8f4c599.jpg]

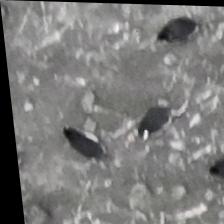

Supplement: Supplementary file 3 — Supplementary Data 1 [file 41467_2023_37132_MOESM3_ESM.zip › train/test_0_5_jpg.rf.29ceb07a47c90e80fda9a50c0bc031dd.jpg]

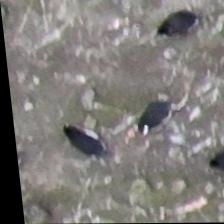

Supplement: Supplementary file 3 — Supplementary Data 1 [file 41467_2023_37132_MOESM3_ESM.zip › train/test_0_5_jpg.rf.2c113c6e0e2c1c3cf75807ff17815740.jpg]

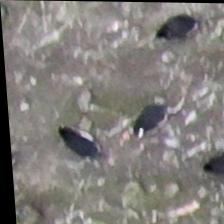

Supplement: Supplementary file 3 — Supplementary Data 1 [file 41467_2023_37132_MOESM3_ESM.zip › train/test_0_5_jpg.rf.512665b0c03a77be57d496b2bf2ca4b4.jpg]

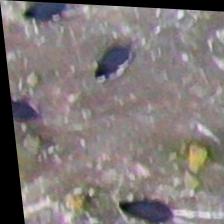

Supplement: Supplementary file 3 — Supplementary Data 1 [file 41467_2023_37132_MOESM3_ESM.zip › train/test_0_7_jpg.rf.881c10891f58a63ebca74e7e784bbcfd.jpg]

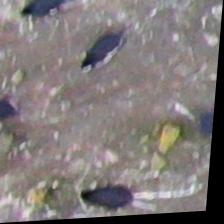

Supplement: Supplementary file 3 — Supplementary Data 1 [file 41467_2023_37132_MOESM3_ESM.zip › train/test_0_7_jpg.rf.8b5ce014d50a7d60b573ddc5b89303e2.jpg]

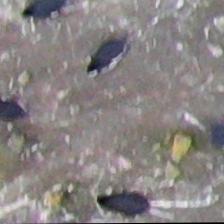

Supplement: Supplementary file 3 — Supplementary Data 1 [file 41467_2023_37132_MOESM3_ESM.zip › train/test_0_7_jpg.rf.93a4afeed85e9feac6892d178f4f0613.jpg]

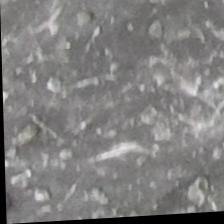

Supplement: Supplementary file 3 — Supplementary Data 1 [file 41467_2023_37132_MOESM3_ESM.zip › train/test_0_9_jpg.rf.733dc037cf6a406624eefa498b9b1566.jpg]

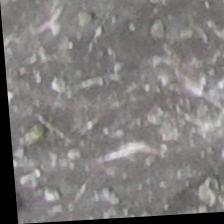

Supplement: Supplementary file 3 — Supplementary Data 1 [file 41467_2023_37132_MOESM3_ESM.zip › train/test_0_9_jpg.rf.8b556eaf400dc82b2daf80a387f6217b.jpg]

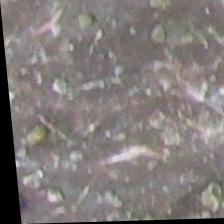

Supplement: Supplementary file 3 — Supplementary Data 1 [file 41467_2023_37132_MOESM3_ESM.zip › train/test_0_9_jpg.rf.e55368737d98a241dfab91bff328102b.jpg]

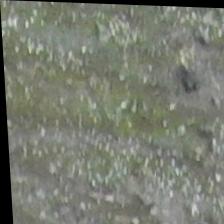

Supplement: Supplementary file 3 — Supplementary Data 1 [file 41467_2023_37132_MOESM3_ESM.zip › train/test_10_0_jpg.rf.a60a14e199b066fb4e7d4f88a01a805b.jpg]

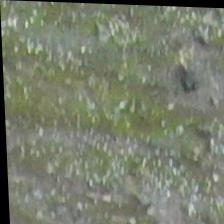

Supplement: Supplementary file 3 — Supplementary Data 1 [file 41467_2023_37132_MOESM3_ESM.zip › train/test_10_0_jpg.rf.b09bddd0ea9a34cc8500771813182664.jpg]

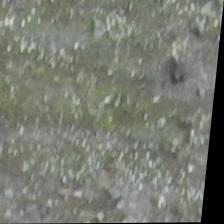

Supplement: Supplementary file 3 — Supplementary Data 1 [file 41467_2023_37132_MOESM3_ESM.zip › train/test_10_0_jpg.rf.f69701e7d806f553c181b163a7ed9c1b.jpg]

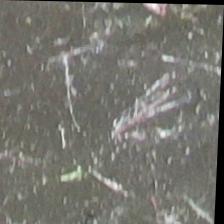

Supplement: Supplementary file 3 — Supplementary Data 1 [file 41467_2023_37132_MOESM3_ESM.zip › train/test_10_10_jpg.rf.2b36ace7bc7eaffc5792a88be79f3588.jpg]

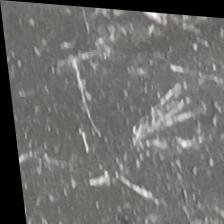

Supplement: Supplementary file 3 — Supplementary Data 1 [file 41467_2023_37132_MOESM3_ESM.zip › train/test_10_10_jpg.rf.826be0dd4b462b543d866fa1d303250e.jpg]

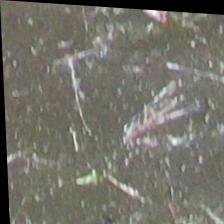

Supplement: Supplementary file 3 — Supplementary Data 1 [file 41467_2023_37132_MOESM3_ESM.zip › train/test_10_10_jpg.rf.c6a75472cfc4a29b5a12a7f6c3c031d9.jpg]

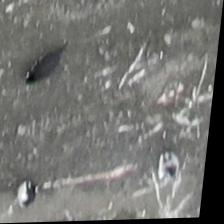

Supplement: Supplementary file 3 — Supplementary Data 1 [file 41467_2023_37132_MOESM3_ESM.zip › train/test_10_11_jpg.rf.85857f86709f4d8dd771d98dfe1c90a1.jpg]

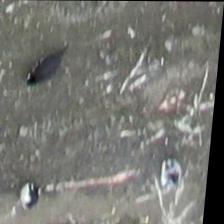

Supplement: Supplementary file 3 — Supplementary Data 1 [file 41467_2023_37132_MOESM3_ESM.zip › train/test_10_11_jpg.rf.9c55b19a9d144f831863024cc7778b1d.jpg]

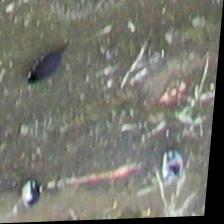

Supplement: Supplementary file 3 — Supplementary Data 1 [file 41467_2023_37132_MOESM3_ESM.zip › train/test_10_11_jpg.rf.ed7a344a3a437dad7993641eb04c9c3a.jpg]

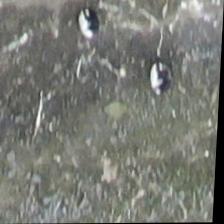

Supplement: Supplementary file 3 — Supplementary Data 1 [file 41467_2023_37132_MOESM3_ESM.zip › train/test_10_12_jpg.rf.7a42b726aa377cc891e7975f0abb6ab2.jpg]

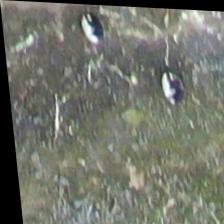

Supplement: Supplementary file 3 — Supplementary Data 1 [file 41467_2023_37132_MOESM3_ESM.zip › train/test_10_12_jpg.rf.dd7dc7799dc543fd3942ea89da61f031.jpg]

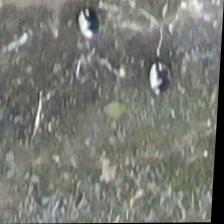

Supplement: Supplementary file 3 — Supplementary Data 1 [file 41467_2023_37132_MOESM3_ESM.zip › train/test_10_12_jpg.rf.ee73f110b584618a5c29eb88288e2e05.jpg]

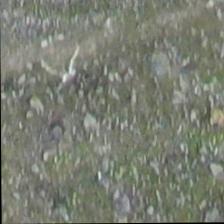

Supplement: Supplementary file 3 — Supplementary Data 1 [file 41467_2023_37132_MOESM3_ESM.zip › train/test_10_13_jpg.rf.4f1b3febb67825f386c1e5f9d0e1697c.jpg]

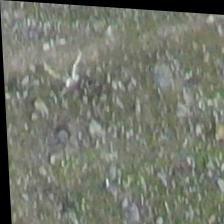

Supplement: Supplementary file 3 — Supplementary Data 1 [file 41467_2023_37132_MOESM3_ESM.zip › train/test_10_13_jpg.rf.89169db8cdf185f9628f321cf6b657c7.jpg]

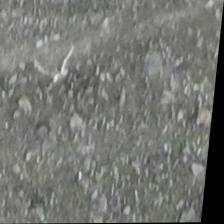

Supplement: Supplementary file 3 — Supplementary Data 1 [file 41467_2023_37132_MOESM3_ESM.zip › train/test_10_13_jpg.rf.930d5e21573a8d783bcfd2d2da470c55.jpg]

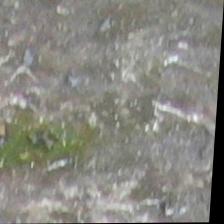

Supplement: Supplementary file 3 — Supplementary Data 1 [file 41467_2023_37132_MOESM3_ESM.zip › train/test_10_2_jpg.rf.2e49d1f3a20c44139d031a6479eefb99.jpg]

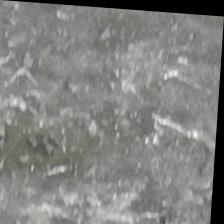

Supplement: Supplementary file 3 — Supplementary Data 1 [file 41467_2023_37132_MOESM3_ESM.zip › train/test_10_2_jpg.rf.528746476a64eda0e8e6f58360f2f172.jpg]

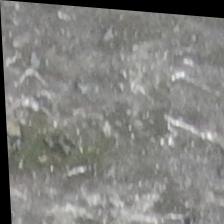

Supplement: Supplementary file 3 — Supplementary Data 1 [file 41467_2023_37132_MOESM3_ESM.zip › train/test_10_2_jpg.rf.f40630ba08ee0370617c46f874ef8f95.jpg]

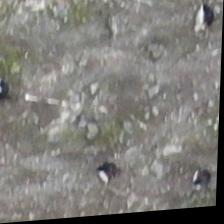

Supplement: Supplementary file 3 — Supplementary Data 1 [file 41467_2023_37132_MOESM3_ESM.zip › train/test_10_4_jpg.rf.afcf2b8bedb7c339424df946c2dcd2c4.jpg]

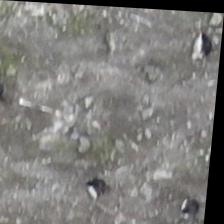

Supplement: Supplementary file 3 — Supplementary Data 1 [file 41467_2023_37132_MOESM3_ESM.zip › train/test_10_4_jpg.rf.e0d78e11cb616b8f48a201cacf162db0.jpg]

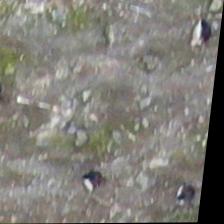

Supplement: Supplementary file 3 — Supplementary Data 1 [file 41467_2023_37132_MOESM3_ESM.zip › train/test_10_4_jpg.rf.e485b7006dcd9713ab4e000e21a5ae55.jpg]

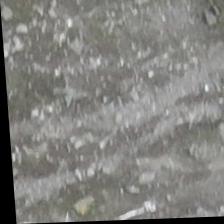

Supplement: Supplementary file 3 — Supplementary Data 1 [file 41467_2023_37132_MOESM3_ESM.zip › train/test_10_5_jpg.rf.26ddc7ab971fb6e4a2d426a92472ede6.jpg]

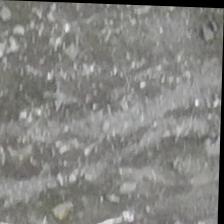

Supplement: Supplementary file 3 — Supplementary Data 1 [file 41467_2023_37132_MOESM3_ESM.zip › train/test_10_5_jpg.rf.a1f9ba0ae11876543510594538ec7b19.jpg]

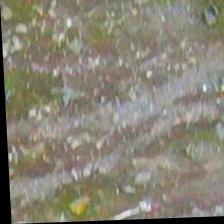

Supplement: Supplementary file 3 — Supplementary Data 1 [file 41467_2023_37132_MOESM3_ESM.zip › train/test_10_5_jpg.rf.feed216aa5fd46db08902e4d03c70cfb.jpg]

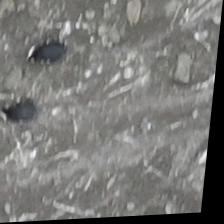

Supplement: Supplementary file 3 — Supplementary Data 1 [file 41467_2023_37132_MOESM3_ESM.zip › train/test_10_7_jpg.rf.0092f453fdbaa7f83a9c62f86c74e5b1.jpg]

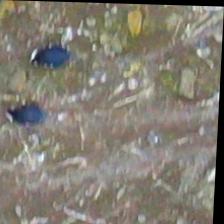

Supplement: Supplementary file 3 — Supplementary Data 1 [file 41467_2023_37132_MOESM3_ESM.zip › train/test_10_7_jpg.rf.21309f7259a3bfe2cba149d24d8b06cd.jpg]

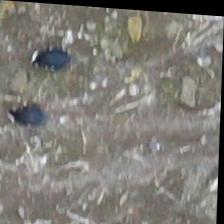

Supplement: Supplementary file 3 — Supplementary Data 1 [file 41467_2023_37132_MOESM3_ESM.zip › train/test_10_7_jpg.rf.cd0fb8e9721d5ea88b89a7f8eefa3627.jpg]

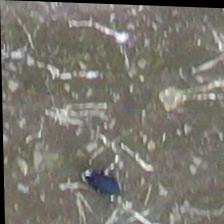

Supplement: Supplementary file 3 — Supplementary Data 1 [file 41467_2023_37132_MOESM3_ESM.zip › train/test_10_9_jpg.rf.77c36725df8de445396d3d37efb660d5.jpg]

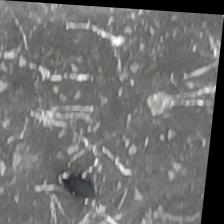

Supplement: Supplementary file 3 — Supplementary Data 1 [file 41467_2023_37132_MOESM3_ESM.zip › train/test_10_9_jpg.rf.a7523e8032346fe1aba884b322113a8a.jpg]

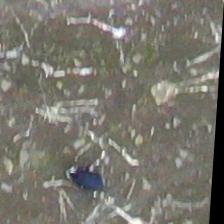

Supplement: Supplementary file 3 — Supplementary Data 1 [file 41467_2023_37132_MOESM3_ESM.zip › train/test_10_9_jpg.rf.b13b5026ad1d700c92030fbc6b3a3796.jpg]

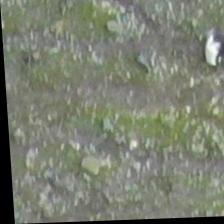

Supplement: Supplementary file 3 — Supplementary Data 1 [file 41467_2023_37132_MOESM3_ESM.zip › train/test_11_0_jpg.rf.c67875127fd716a96743857dcc4dd59d.jpg]

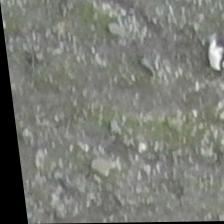

Supplement: Supplementary file 3 — Supplementary Data 1 [file 41467_2023_37132_MOESM3_ESM.zip › train/test_11_0_jpg.rf.f5851e1ac6dd63ce96e9059f87b36ada.jpg]

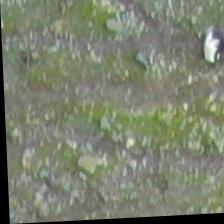

Supplement: Supplementary file 3 — Supplementary Data 1 [file 41467_2023_37132_MOESM3_ESM.zip › train/test_11_0_jpg.rf.f9c1a40b4110f5e1f5542a1c3f1e8110.jpg]

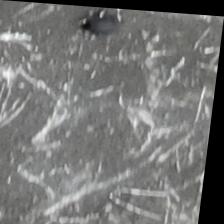

Supplement: Supplementary file 3 — Supplementary Data 1 [file 41467_2023_37132_MOESM3_ESM.zip › train/test_11_10_jpg.rf.015ca52ade3981861d6ca17be325203b.jpg]

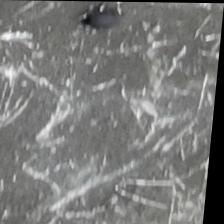

Supplement: Supplementary file 3 — Supplementary Data 1 [file 41467_2023_37132_MOESM3_ESM.zip › train/test_11_10_jpg.rf.5a191e1812189f1d7833c4761fd647b6.jpg]

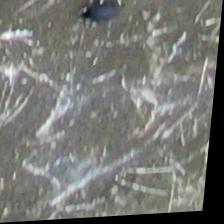

Supplement: Supplementary file 3 — Supplementary Data 1 [file 41467_2023_37132_MOESM3_ESM.zip › train/test_11_10_jpg.rf.ee02cec6e876aa6935c913b1403bce26.jpg]

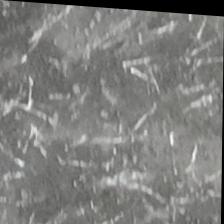

Supplement: Supplementary file 3 — Supplementary Data 1 [file 41467_2023_37132_MOESM3_ESM.zip › train/test_11_11_jpg.rf.695c54a22b9a8ece5d4d439f0a543095.jpg]

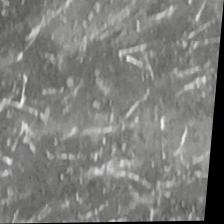

Supplement: Supplementary file 3 — Supplementary Data 1 [file 41467_2023_37132_MOESM3_ESM.zip › train/test_11_11_jpg.rf.7c8459e6855f4bbd39b75b85f0d73ad4.jpg]

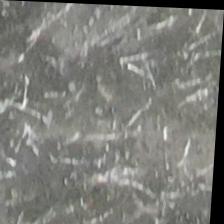

Supplement: Supplementary file 3 — Supplementary Data 1 [file 41467_2023_37132_MOESM3_ESM.zip › train/test_11_11_jpg.rf.93e6732b4ab4991de17a38539980c55e.jpg]

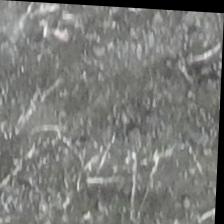

Supplement: Supplementary file 3 — Supplementary Data 1 [file 41467_2023_37132_MOESM3_ESM.zip › train/test_11_12_jpg.rf.62a647cd55eb15f937a4ced6ed432b24.jpg]

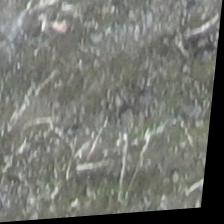

Supplement: Supplementary file 3 — Supplementary Data 1 [file 41467_2023_37132_MOESM3_ESM.zip › train/test_11_12_jpg.rf.69b7028a771ea983831045859d05723f.jpg]

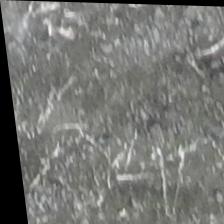

Supplement: Supplementary file 3 — Supplementary Data 1 [file 41467_2023_37132_MOESM3_ESM.zip › train/test_11_12_jpg.rf.6cad88437f317ed0ae80e8fbd7002a65.jpg]

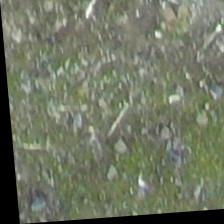

Supplement: Supplementary file 3 — Supplementary Data 1 [file 41467_2023_37132_MOESM3_ESM.zip › train/test_11_13_jpg.rf.0d5c1e63a5884ce30668b9db7db5c6ef.jpg]

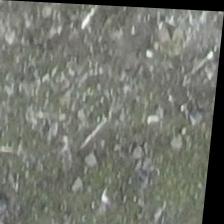

Supplement: Supplementary file 3 — Supplementary Data 1 [file 41467_2023_37132_MOESM3_ESM.zip › train/test_11_13_jpg.rf.8ac53e13606955a9787ef6a272aa9e26.jpg]

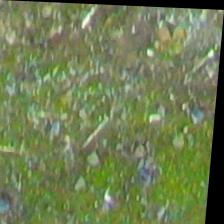

Supplement: Supplementary file 3 — Supplementary Data 1 [file 41467_2023_37132_MOESM3_ESM.zip › train/test_11_13_jpg.rf.e0f21d03a572e1d045c300e534ec14ac.jpg]
