# Supplementary material for: The contribution of penguin guano to the Southern Ocean iron pool
Source: Nat Commun. 2023 Apr 11;14:1781. doi: 10.1038/s41467-023-37132-5 (PMC10090129; doi:10.1038/s41467-023-37132-5)
Supplement: Supplementary file 5 — Reporting Summary [file 41467_2023_37132_MOESM5_ESM.pdf]

nature portfolio

Corresponding author(s): Oleg Belyaev

Last updated by author(s): Jan 16, 2023

Reporting Summary

Nature Portfolio wishes to improve the reproducibility of the work that we publish. This form provides structure for consistency and transparency in reporting. For further information on Nature Portfolio policies, see our Editorial Policies and the Editorial Policy Checklist.

Statistics

For all statistical analyses, confirm that the following items are present in the figure legend, table legend, main text, or Methods section.

n/a	Confirmed	
☐	☒	The exact sample size (*n*) for each experimental group/condition, given as a discrete number and unit of measurement
☐	☒	A statement on whether measurements were taken from distinct samples or whether the same sample was measured repeatedly
☒	☐	The statistical test(s) used AND whether they are one- or two-sided<br>*Only common tests should be described solely by name; describe more complex techniques in the Methods section.*
☒	☐	A description of all covariates tested
☒	☐	A description of any assumptions or corrections, such as tests of normality and adjustment for multiple comparisons
☐	☒	A full description of the statistical parameters including central tendency (e.g. means) or other basic estimates (e.g. regression coefficient) AND variation (e.g. standard deviation) or associated estimates of uncertainty (e.g. confidence intervals)
☒	☐	For null hypothesis testing, the test statistic (e.g. *F*, *t*, *r*) with confidence intervals, effect sizes, degrees of freedom and *P* value noted<br>*Give P values as exact values whenever suitable.*
☒	☐	For Bayesian analysis, information on the choice of priors and Markov chain Monte Carlo settings
☒	☐	For hierarchical and complex designs, identification of the appropriate level for tests and full reporting of outcomes
☒	☐	Estimates of effect sizes (e.g. Cohen's *d*, Pearson's *r*), indicating how they were calculated

Our web collection on statistics for biologists contains articles on many of the points above.

Software and code

Policy information about availability of computer code

Data collection	Training, evaluation and testing datasets for deep-learning model were created (annotated, augmented and analysed) using Roboflow online platform. Dataset is available on https://github.com/obkorolev/penguin_iron_paper
Data analysis	Datasets were used to train deep-learning model for object detection. Hyperparameters of the model can be foun on https://github.com/obkorolev/penguin_iron_paper. The model was obtained from https://github.com/tensorflow/models.<br>Mosaics of Vapour Col were generated using Agisoft Metashape Professional version 1.8.3 build 14331 (64 bit).<br>Training and evaluation tasks were performed using the TensorFlow 2.0 machine learning platform by Google.

For manuscripts utilizing custom algorithms or software that are central to the research but not yet described in published literature, software must be made available to editors and reviewers. We strongly encourage code deposition in a community repository (e.g. GitHub). See the Nature Portfolio guidelines for submitting code & software for further information.

Data

Policy information about availability of data

All manuscripts must include a data availability statement. This statement should provide the following information, where applicable:

- Accession codes, unique identifiers, or web links for publicly available datasets
- A description of any restrictions on data availability
- For clinical datasets or third party data, please ensure that the statement adheres to our policy

The training dataset and iron concentrations data generated during the current study are provided in the Supplementary Materials.

Human research participants

Policy information about studies involving human research participants and Sex and Gender in Research.

Reporting on sex and gender	N/A
Population characteristics	N/A
Recruitment	N/A
Ethics oversight	N/A

Note that full information on the approval of the study protocol must also be provided in the manuscript.

Field-specific reporting

Please select the one below that is the best fit for your research. If you are not sure, read the appropriate sections before making your selection.

☐ Life sciences ☐ Behavioural & social sciences ☒ Ecological, evolutionary & environmental sciences

For a reference copy of the document with all sections, see nature.com/documents/nr-reporting-summary-flat.pdf

Ecological, evolutionary & environmental sciences study design

All studies must disclose on these points even when the disclosure is negative.

Study description

In this study, breeding site guano volumes estimated from drone images, deep learning-powered penguin census, and guano chemical composition were used to assess the iron export to the Antarctic waters from the Chinstrap penguin (Pygoscelis antarcticus). For guano volume estimation, guano areas as viewed from an UAV were calculated and the volumes were computed, obtaining subsequently the Fe amount present in the guano, using Fe concentrations found in guano samples of the area. To cross-validate the results, the population of Chinstrap penguins at a specific time was obtained, to then estimate the guano production based on their daily depositions. The amount of Fe present in the colony was compared to the Fe concentration found in the waters surrounding the colony, obtaining thus the estimation, specific to time and location, of the Fe release from the colony.

Research sample

Guano samples collection (n=23) from three different substrates to explore possible variability in sample sources: from soil, ice and from a specific guano collector. The latter one ensured a clean, soil-free sample colection. Samples were collected from both from guano rich and guano poor areas within the colony (except "trap", which needed penguin presence to obtain direct depositions), to ensure spatial heterogeneity of the samples.
Drone imagery was collected over Vapour Col colony at different heights to ensure the coverage of the maximum possible area, based on the available hardware.

Sampling strategy

Fieldwork consisted on guano samples collection (n=23) from three different substrates: randomly from soil and ice and from a specific guano collector placed in the colony 24 hours prior to sampling, to allow penguins to get used to the presence of the trap and therefore maximize the possibilities of deposition collection.

Drone imagery was collected following a sweeping flying route to allow the coverage of most of the extension of the colony, with battery limitations. The flight was performed using DJI Mavic 2 Pro UAV, captured from a flight height of 30 m and speed of 4.9 m s-1.

Data collection

Guano samples collection: In January and February 2021, fresh guano samples were collected from the Vapour Col (VC) breeding site. To ensure the collection of uncontaminated samples, the guano was differentiated according to the substrate from which it was taken and three types of samples were analysed: soil, ice, and guano collected in a “trap”. The traps, made of a polyethylene plastic plate with a PVC frame measuring 40x30 cm, were placed in the colony for 24 hours before sampling. The fresh VC guano samples (n = 23) were then collected manually with a plastic spoon and stored in either polyethylene bags or acid-cleaned vials and kept frozen at -20 °C until analysis.

Photographical data collection: On February 8, 2021, 377 RGB images of the Northern Tip of Vapour Col were taken with the help of

an unmanned aerial vehicle (UAV). The images had a resolution of 3,000×4,000 pixels and were captured from a flight height of 30 m and speed of 4.9 m s-1. The UAV used was a DJI Mavic 2 Pro, equipped with an RGB sensor (Hasselblad Camera). DJI's Ground Station Pro photogrammetric flight planning software was utilized to configure the flights, and Agisoft Metashape Professional photogrammetric software was used to generate an orthomosaic from the photographs.

Timing and spatial scale

The field data collection took place from January 28, to February 8, 2021 for guano samples, based on campaign schedules and February 8, 2021 for drone footage, due to the presence of clear sky and favourable meteorology in Vapour Col Chinstrap penguin rookery, Deception Island, South Shetland Islands, Antarctica.

Data exclusions

Neither of guano samples or drone footage data were exluded in this study. Refer to Methods section for detailed explanation about data processing.

Reproducibility

This study provides detailed information that, with the code, training data, and iron concentration in guano samples provided as Supplementary Data, allow this study to be replicated if desired. When performing object detection model training, the tunning of the hyperparameters can produce different outputs. The hyperparameters configuration used in this study are provided in the GitHub repository (view Code Availability statement).

Randomization

Samples were randomly collected from soil (n=12) and ice (n=6) substrates. Guano collectors were placed randomly in areas with highest penguin denisties (avoiding disturbance) to ensure sample collection (n=5). The acquired samples were subsequently divided into three groups based on their provenance: soil, ice, and trap.

Blinding

Blinding was not used for sample collection as samples specifically were required to be from three available substrates present in the region, which however were randomly acquired. All data was used for further analysis.

Did the study involve field work? [X] Yes [ ] No

Field work, collection and transport

Field conditions

During sample collection air temperature ranged from 0ºC to 4ºC and occasionally rained and snowed.

Location

Vapour Col Colony (62°59'29" S, 60°43'32" W), Deception Islands, South Shetland Islands. The water depth at the sampling point was estimated to be 2 m.

Access & import/export

The access to Deception Island was granted by the Spanish Polar Committee (SPC), which was accessed on board of the Sarmiento de Gamboa oceanographic vessel. Access to the Vapour Col colony for sampling tasks was granted by the SPC during the sampling period (January-February). All samples were transported to Spain under the authorization of the SPC.

Disturbance

To minimize disturbance of penguins caused by drones, the minimum flying height was 30 m, which, according to existing literature, represents the minimum height where no disturbance effects on most terrestrial seabirds were observed.

Reporting for specific materials, systems and methods

We require information from authors about some types of materials, experimental systems and methods used in many studies. Here, indicate whether each material, system or method listed is relevant to your study. If you are not sure if a list item applies to your research, read the appropriate section before selecting a response.

Materials & experimental systems

n/a	Involved in the study	
[X]	[ ]	Antibodies
[X]	[ ]	Eukaryotic cell lines
[X]	[ ]	Palaeontology and archaeology
[ ]	[X]	Animals and other organisms
[X]	[ ]	Clinical data
[X]	[ ]	Dual use research of concern

Methods

n/a	Involved in the study	
[X]	[ ]	ChIP-seq
[X]	[ ]	Flow cytometry
[X]	[ ]	MRI-based neuroimaging

Animals and other research organisms

Policy information about studies involving animals; ARRIVE guidelines recommended for reporting animal research, and Sex and Gender in Research

Laboratory animals

The study did not involve laboratory animals

Wild animals	Aerial footage was taken using dron over the colony, flying at a minimum 30 m height, thus avoiding disturbance to the penguins.
Reporting on sex	Findings do not apply to a specific sex. Guano samples and aerial footage covered both penguin genders of a wide spectrum of development stages. Permissions for guano and drone footage data collection were granted by the Spanish Polar Commitee.
Field-collected samples	Guano samples collection (n=23) from three different substrates: from soil, ice and from a specific guano collector. Drone imagery collected over the Vapour Col colony at different heights.
Ethics oversight	All sampling procedures were performed under the authorization of the Spanish Polar Committee

Note that full information on the approval of the study protocol must also be provided in the manuscript.
